# A New Molecular Phylogeny of Salps (Tunicata: Thalicea: Salpida) and the Evolutionary History of Their Colonial Architecture

**DOI:** 10.1093/iob/obad037

**Published:** 2023-09-27

**Authors:** A Damian-Serrano, M Hughes, K R Sutherland

**Affiliations:** University of Oregon, Department of Biology, Institute of Ecology and Evolution. 473 Onyx Bridge, 5289 University of Oregon, Eugene OR 97403-5289, USA; P.O. Box 4979, Kailua Kona HI 96745, USA; University of Oregon, Department of Biology, Institute of Ecology and Evolution. 473 Onyx Bridge, 5289 University of Oregon, Eugene OR 97403-5289, USA

## Abstract

Salps are marine pelagic tunicates with a complex life cycle, including a solitary and colonial stage composed of asexually budded individuals. These colonies develop into species-specific architectures with distinct zooid orientations, including transversal, oblique, linear, helical, and bipinnate chains, as well as whorls and clusters. The evolutionary history of salp colony architecture has remained obscured due to the lack of an ontology to characterize architectures, as well as a lack of phylogenetic taxon sampling and resolution of critical nodes. We (1) collected and sequenced eight species of salps that had never been sequenced before, (2) inferred the phylogenetic relationships among salps, and (3) reconstructed the evolutionary history of salp colony architecture. We collected salp specimens via offshore SCUBA diving, dissected tissue samples, extracted their DNA, amplified their 18S gene, and sequenced them using Sanger technology. We inferred the phylogeny of Salpida based on 18S using both Maximum Likelihood and Bayesian approaches. Using this phylogeny, we reconstructed the ancestral states of colony architecture using a Bayesian ordered Markov model informed by the presence and absence of specific developmental mechanisms that lead to each architecture. We find that the ancestral salp architecture is either oblique or linear, with every other state being derived. Moreover, linear chains have evolved independently at least three times. While transversal chains are developmentally basal and hypothesized to be ancestral, our phylogenetic topology and reconstructions strongly indicate that they are evolutionarily derived through the loss of zooid torsion. These traits are likely critical to multijet locomotory performance and evolving under natural selection. Our work showcases the need to study the broader diversity of salp species to gain a comprehensive understanding of their organismal biology, evolutionary history, and ecological roles in pelagic ecosystems.

## Abstract (es)

Spanish Abstract

Las salpas son tunicados pelágicos marinos con un ciclo vital complejo que incluye una fase solitaria y una colonial, compuesta de individuos gemados asexualmente. Al desarrollarse, estas colonias forman arquitecturas diferentes según la especie con orientaciones distintivas de los zooides. Estas arquitecturas incluyen las cadenas transversales, las oblícuas, las lineales, las solenoides, y también las formas de rueda y conglómero. La historia evolutiva de la arquitectura colonial de las salpas ha permanecido en la sombras debido a la falta de un ontología para caracterizar estas arquitecturas, además de por falta de representación taxonómica y resolución de nodos críticos en la filogenia. Hemos (1) muestreado y secuenciado ocho especies de salpa que nunca antes habían sido secuenciadas, (2) hemos inferido las relaciones filogenéticas entre salpas, y (3) reconstruido la historia evolutiva de la arquitectura colonial en salpas. Hemos obtenido especímenes de salpas mediante buceo con escafandra en alta mar, diseccionado muestras de tejido, extraído el ADN, amplificado y secuenciado el gen ribosomal 18S mediante tecnología Sanger. Hemos inferido la filogenia del orden Salpida en base al gen 18S utilizando tanto Máxima Verosimilitud como métodos Bayesianos. Utilizando esta filogenia, hemos reconstruido los estados ancestrales de la arquitectura colonial mediante un modelo Bayesiano Markov ordinal informado por la presencia y ausencia de mecanismos ontogenéticos que derivan en cada arquitectura. Hallamos que la arquitectura ancestral en las salpas fue o bien oblícua o lineal, con todos los demás estados derivados. Además, las cadenas lineales han evolucionado de forma independiente por lo menos tres veces. Las cadenas transversales son el estado basal en el desarrollo y han sido hipotetizadas como el estado ancestral. Sin embargo, nuestra topología filogenética y reconstrucciones indican firmemente que son en realidad derivadas a través de la pérdida del mecanismo de torsion de los zooides. Es probable que estos rasgos morfológicos sean críticos para la eficiencia locomotora de la propulsión multi-chorro y por tanto hayan evolucionado bajo el efecto de la selección natural. Nuestro trabajo demuestra la necesidad de estudiar la amplia diversidad de especies en las salpas para obtener una comprensión integral de su biología organísmica, historia evolutiva, y roles ecológicos en los ecosistemas pelágicos.

## Introduction

Salps (Chordata: Tunicata: Thaliacea: Salpida) are marine pelagic tunicates that filter-feed on microbial plankton. Salp life cycles consist of a solitary stage (oozooid) that asexually buds aggregate colonies of blastozooids, which can sexually reproduce, and brood oozooids in their placenta (Bone 1998). Salp colonies are budded in double chains of zooid pairs with mirror (chiral) symmetry. These colonies develop into a wide variety of architectures across species, including transversal chains, oblique chains, linear chains, whorls, clusters, and helical solenoids (Damian-Serrano & Sutherland 2023a; Madin 1990). These architectures present different relative orientations of the individual zooids relative to each other and to the colony elongation axis (Madin 1990).

**Fig. 1.**
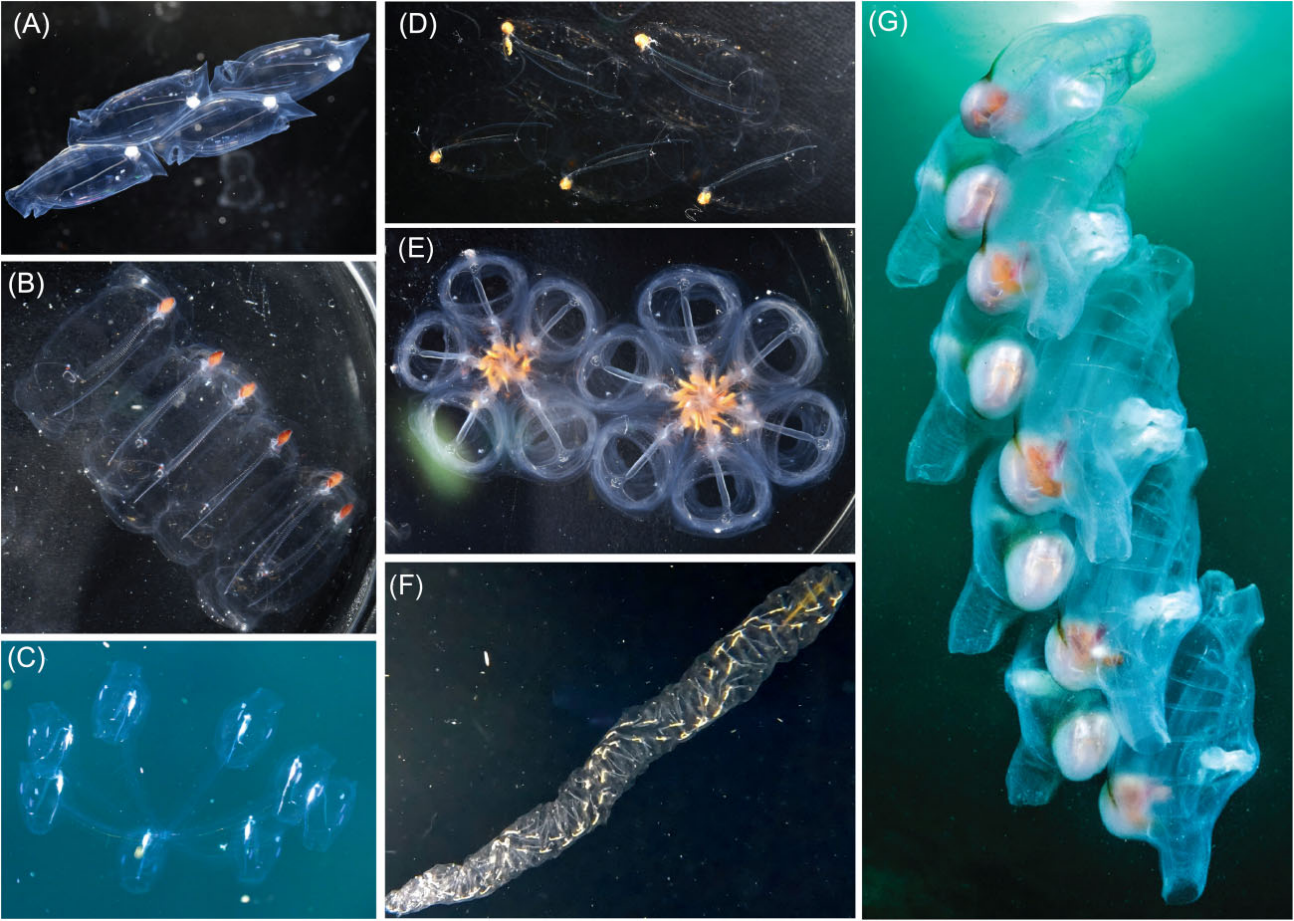
Salp colonies representing every colonial architecture we observed across salp species. A. Linear chain (*Soestia zonaria*), B. transversal chain (*Pegea* sp.), C. cluster (*Cyclosalpa sewelli*, video frame by Brad Gemmell), D. bipinnate chain (*Ritteriella retracta*), E. whorls (*Cyclosalpa affinis*), F. helical chain (*Helicosalpa virgula*), and G. oblique chain (*Thetys vagina*, photograph by Patrick Webster).

**Fig. 2.**
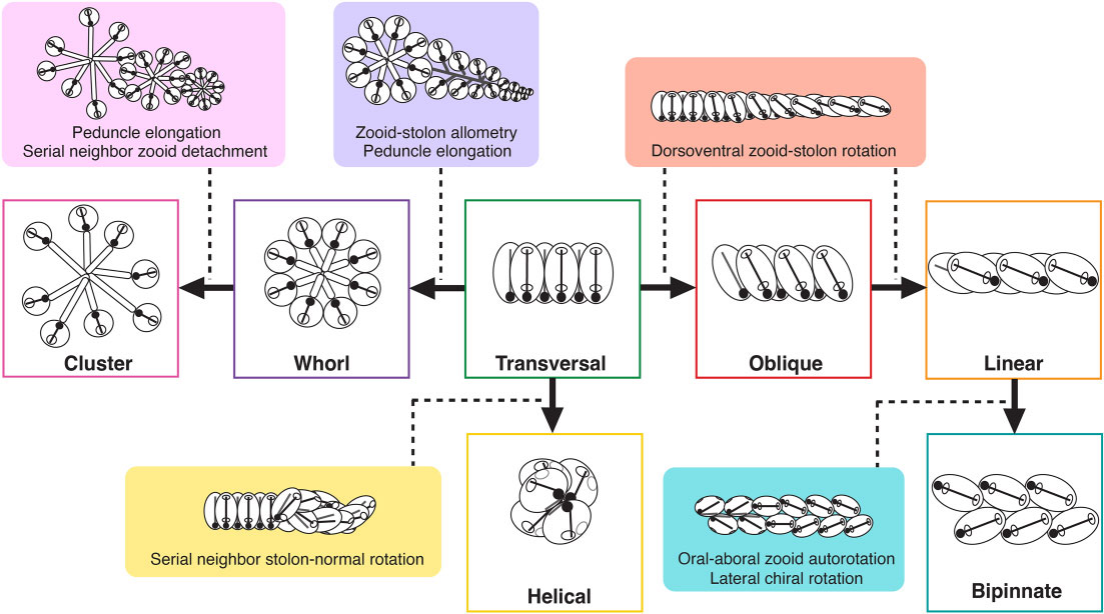
Developmental pathway model of salp colony architectures from Damian-Serrano & Sutherland (2023a), with the transversal architecture representing the earliest developmental stage of every species as well as in the adult stage of some species.

The diversity of salp colony architectures (Fig. 1) is distributed across 41 species of salps (Madin 1990), but the phylogenetic distribution and evolutionary history remain unknown (Damian-Serrano & Sutherland 2023a). The main challenges in reconstructing this history have been two-fold: first, the lack of a hypothesis framework for comparing homologies and understanding differences in their structure, and second, the lack of a phylogenetic tree that includes taxa from every architecture and from every described lineage where it has evolved. The first challenge comes from how the arrangement and relative orientation of blastozooids in different colony architectures present a three-dimensional problem, where the axes and angles of reference shift in ways that are challenging to compare. All blastozooid colonies are budded as transversal double chains and then develop into the different colonial architectures we observe across the diversity of salp species. Damian-Serrano & Sutherland (2023a) leveraged the similarity of this developmental stage as a baseline to define planes of observation and reference, from which we can examine deviations in angles, establish the series of gains and losses of transformation mechanisms that determine the distinct developmental pathways, and identify homologies between extant adult terminal stages of some species and intermediate stages in the development of other species (Fig. 2). Damian-Serrano & Sutherland (2023a) tracked the shared developmental stages in colony reconfiguration, which provided preliminary evidence on the homology relationships between architectural states. This hierarchical, developmentally informed, characterization of architectural variation provides a grounding in homology that a flat distribution of categorical states does not. The second challenge relates to phylogenetic node resolution and taxon sampling. Govindarajan et al. (2011) used 18S sequencing to create the first comprehensive phylogenetic tree of Thaliacea (second after the tunicate phylogeny presented in Tsagkogeorga et al. 2009), including 20 salp species. However, it could not provide a complete picture of how salp colony architecture evolved since the phylogenetic placements of *Pegea* (transversal architecture) and *Thalia* (one of two lineages where oblique architectures are found) had low statistical support; and *Helicosalpa* spp. (helical architecture) had never been sequenced. Moreover, several other morphologically unique salp species with known colony architectures (such as *Metcalfina hexagona* or *Ihlea punctata*) remained to be sequenced and placed on a phylogenetic tree and may be representatives of under-sampled lineages. Madin (1990) hypothesized that the lineage containing salps with transversal architectures such as *Pegea* is sister to all other salps, that the most recent common ancestor (MRCA) of salps is also transversal, and that *Pegea* species (as well as the elusive species *Traustedtia multitentaculata*) thus retain this ancestral character. Govindarajan et al. (2011) discuss similar ideas from Madin (1974), including that whorl and transversal chain architectures are closer to the ancestral form while linear chains are the most derived.

In addition to a robust phylogeny with ample taxon sampling, we need a realistic model of character evolution to accurately reconstruct the evolutionary history of colonial architecture in salps. A reliable evolutionary model should be based on external biological information about the nature of the character in hand, its homologies, and development (Wagner et al. 2000). From the developmental ontology in Damian-Serrano & Sutherland (2023a), every architecture can be understood as a product of one or more specific developmental transformation mechanisms. Moreover, some adult colony architectures are conceptualized as intermediary stages in the development of other architectures (Fig. 2), where only a partial transformation occurred (i.e., oblique chains with zooids partially rotated, between a transversal and a linear chain form), or where further developmental transformations build upon previous ones (i.e., clusters derive from whorls with subsequent separation of serial neighbors and elongation of peduncles). This developmental ontology suggests that ordered or structured Markov models, where some character state gains depend on the loss of others (Tarasov 2019), would be a realistic approximation to the mode of evolution of colony architecture, and thus can serve as a model for an evolutionary ontology, where new architectural states can only arise from the gain and loss of developmental transition mechanisms. Alternatively, we hypothesize that the dorsoventral zooid-stolon angle drives the primary differentiation between transversal-like (transversal, whorl, and cluster), oblique, and linear-like (linear and bipinnate) architectures could be conceptualized as a continuous character with a gradual mode of evolution along the branches.

Here, we (1) collected and sequence eight species of salps for the first time, (2) inferred the phylogenetic relationships among salps using the 18S gene through Maximum Likelihood (ML) and Bayesian approaches, and (3) reconstructed the evolutionary history of salp colony architecture using Bayesian ordered Markov models (OMMs) and continuous character models.

## Materials and methods

### Obtaining 18S gene sequences for phylogenetic analysis

To build a well-resolved molecular phylogeny, we primarily used the 18S gene accession list for salps and outgroups from Govindarajan et al. (2011) with a few modifications. First, we suspect that the accession number HQ015280.1 (uncultured bacterium) is a typo for HQ015380.1 *Pyrosomella verticillata*, and thus used the latter instead. Second, the authors from Govindarajan et al. (2011) state to have included a sequence for the ascidian *Halocynthia igaboja* but the accession number is not reported, so we searched GenBank and found accession AY903925.1 for this species, which we used. Third, we included an additional outgroup 18S sequence AJ250778.1 for *Ciona intestinalis*, which helped stabilize many nodes. Fourth, we included four new salp accessions found in Genbank representing the species *Brooksia lacromae, Thalia longicauda*, and *Salpa younti*, which were not available at the time of the Govindarajan et al. (2011) study. Finally, we expanded taxon sampling by collecting tissue samples from understudied (not sequenced before) salp species (*M. hexagona, I. punctata, Helicosalpa virgula, Helicosalpa younti, Cyclosalpa bakeri, Cyclosalpa pinnata*, and *Ritteriella amboinensis*) using tissue samples from specimens we collected while bluewater SCUBA diving (Haddock & Heine 2005) from a small vessel off the coast of Kailua-Kona (Hawai'i Big Island, 19°42“38.7“ N 156°06”15.8” W), over 2000 m of offshore water. When possible, we sampled a variety of tissues from the zooid, excluding the gut to avoid contamination from food particles, as well as the tunic to avoid clogging the DNA extraction columns. These samples were preserved in ethanol at room temperature until the point of DNA extraction in the lab. We included species representing every salp genus with the exception of *Traustedtia*. A list of accession numbers for all the sequences used in this study is available in Supplementary Table 1.

In order to obtain new 18S sequences from the tissue samples we collected, we extracted DNA and amplified the 18S gene using the following protocol. We digested the tissue samples with proteinase K at 56∘C for 1–2 h after rehydrating them in nuclease-free water for 2 min and used the DNeasy Blood & Tissue kit (Qiagen, Hilden, Germany) to extract DNA, eluting twice at 56∘C for 10 min to a final volume of 50 μl. Then, we evaluated extraction yields using Qubit 2.0 in the High Sensititvity (HS) range. To reduce Polymerase Chain Reaction (PCR) inhibition from co-extracted compounds in salp tissues, we diluted these extracts 1:10 in water. This dilution was necessary to successfully perform PCR from these materials. We amplified the 18S gene from these templates using the universal animal primers designed in Damian-Serrano et al. (2022) 18S 400–420 5’ AAC GGC TAC CAC ATC CAA GG 3’, 18S 1651–1675 5’ CCT TGT TAC GAC TTT TAC TTC CTCT 3’. For each 20 μl reaction volume, we used 1 μl of diluted extraction template, 0.5 μl of each primer (10 μM), 3 μl of BSA (20 μg/μl), 10 μl of 2X PCR Mastermix (Thermo Scientific, USA), and 5μl of water. This higher than usual concentration of Bovine Serum Albumin (BSA) was necessary to reduce the inhibitory effect of coextracted compounds in the extraction template. The thermal cycles included an initial denaturation at 95∘C for 2 min, followed by 30 cycles of denaturation at 95∘C for 25 s, annealing at 54°C for 25 s, and elongation at 72∘C for 2 min, followed by final elongation at 72∘C for 10 min. Each batch of reactions included a negative control using the AE elution buffer used in extraction. We then visualized the PCR products using gel electrophoresis (1.5–2% agarose gel dyed with SYBR Safe DNA Stain and purple loading dye) to check for amplification. Those PCR products that showed a distinct band in the gel were then purified using Omega Mag-Bind magnetic beads or Zymo DNA Clean & Concentrator-5 (Zymo Research) and quantified using a Qubit 2.0 fluorometer (Thermo Fisher Scientific, USA).

In order to sequence these purified amplicons, we relied on Sanger sequencing. First, we aliquoted the purified amplicons into two sets, with the addition of 12 picomoles of forward and reverse primers, respectively. These samples were sent to the Center for Quantitative Life Sciences at Oregon State University for sequencing. The forward and reverse chromatographs of each sample were trimmed to an error probability limit of 0.05 and assembled *de novo* them using Geneious Prime (version 2023.0.4) software.

### Phylogenetic inference

**Fig. 3.**
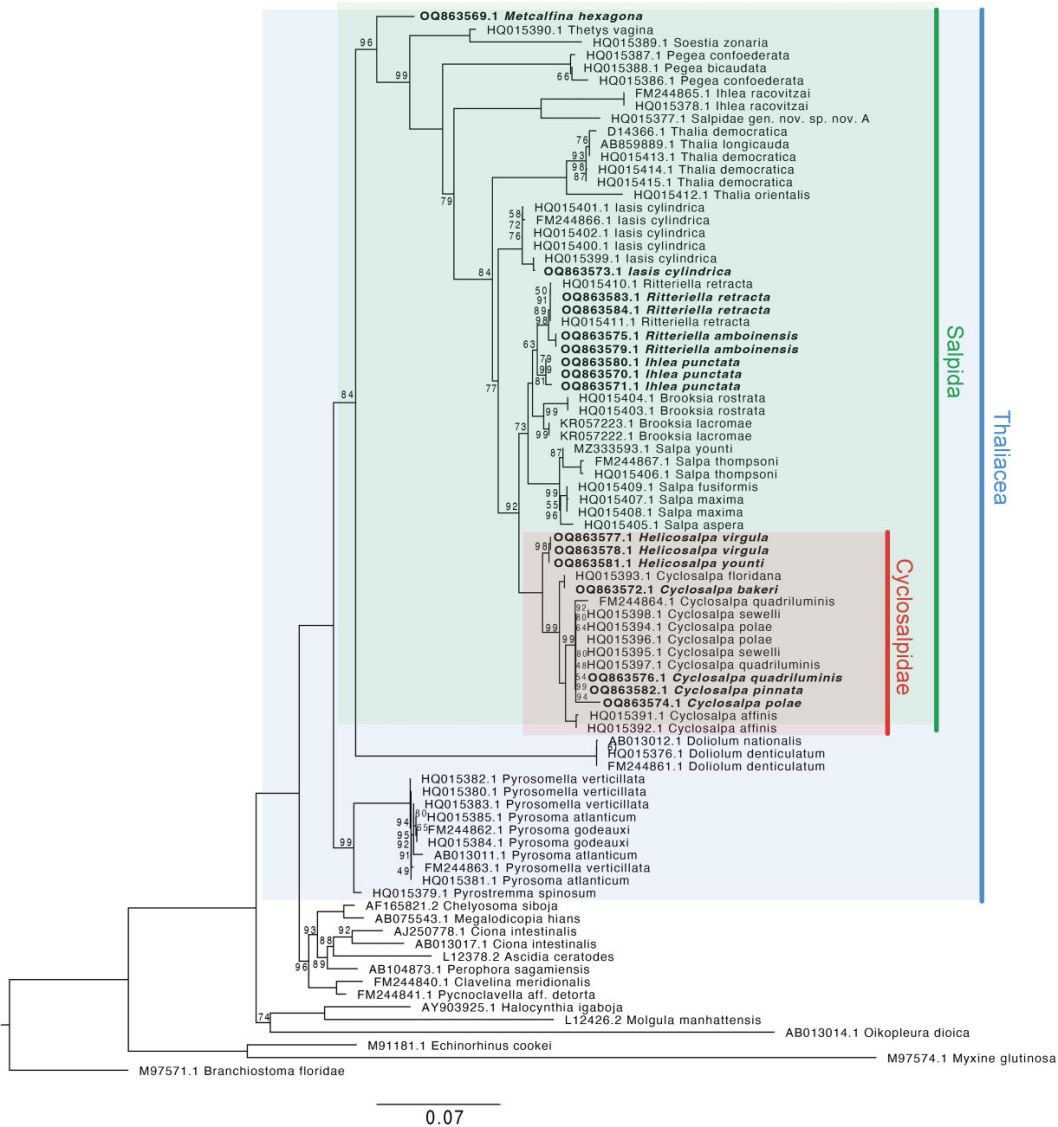
Maximum Likelihood phylogeny. Nodes labeled with bootstrap support. Unlabeled nodes have bootstrap support of 100. Tip labels in bold font are new additions from this study.

We aligned these sequences using MUSCLE 5.1 (Edgar 2004) with default settings. As a sensitivity analysis, we alternatively aligned them with MAFFT 7.419 (Katoh et al. 2009) with default settings. In addition, we experimented with post-processing these alignments with GBLOCKS 0.91b (Castresana 2000) with default settings except for allowing half-gap positions (as used in Govindarajan et al. 2011). The alignment contained every sequence except for *C. bakeri* specimen D27-Cbak-B-1, which appeared truncated and non-comparable after post-processing. GBLOCKS retained 43% of sites when aligning with MUSCLE and 45% of sites when aligning with MAFFT. To make an ML inference from these alignments, we used IQTree 1.6.12 (Nguyen et al. 2015) with 1000 bootstrap replicates (Fig. 3). Node support was reported using bootstrap support (BS). The consensus trees obtained using the model selected by the best Bayesian Information Criterion (TIM3e + R5: transition model with equal base frequency, with five rate categories) and using GTR + I + Gamma are congruent, regardless of whether MAFFT or MUSCLE was used for alignment. However, the consensus trees with GBLOCKS were not congruent with these trees by several nodes which had low support, due to many trimmed sequences appearing identical. We suspect that GBLOCKS is removing critical phylogenetic signal from the data, and therefore decided not to use it for downstream analyses. All phylogenetic tree files and sequence alignments are available in the Dryad repository (Damian-Serrano & Sutherland 2023b).

To infer a Bayesian phylogeny, we used RevBayes 1.0.10 (Höhna et al. 2016) with a GTR + I + Gamma model on the MUSCLE alignment. The Bayesian topology analysis converged across all parameters and the consensus tree was congruent with the ML trees, though some shallow nodes (comprising individual genera which bear very short branches in the ML tree) were unresolved as polytomies (SM Fig. 1). Node support was reported using posterior probabilities (PP). All trees were rooted post-inference using *Branchiostoma floridae* (a representative of Cephalochordata) as the known sister tip to all other taxa based on published phylogenomic analyses (Dunn et al. 2008). In order to examine the evolution of traits on the salp species phylogeny, we built an ultrametric time tree using a relaxed molecular clock in RevBayes with uncorrelated lognormal rates under a GTR + I + Gamma process. We constrained the topology to be congruent with the ML consensus tree. The ML tree was used instead of the Bayesian tree to provide the topology constraint because the former presents no polytomies. This constraint tree was rooted and made ultrametric using the *chronos* function in the R package *ape* 5.6 (Paradis & Schliep 2019). The resulting time tree (SM Fig. 2) was subsequently pruned to remove non-salp outgroups and the undescribed salp species ingroup. Further, we pruned the tree to retain a single representative of each species. In the cases where the species appear as paraphyletic, we choose to drop the species duplicates that maximize branch lengths between species, since we hypothesize that specimens that are more nested with another species are more likely to represent hybrids or misidentifications within the genus.

### Mapping salp colony architecture

We hand-collected between one and five specimens of adult blastozooid colonies from each target species in 1-liter jars via bluewater SCUBA diving (Haddock & Heine 2005). Within 12 h of collection, we took photographs of these live colonies using a Nikon DSLR camera with a 75 mm lens facing downwards on a tripod with the colonies fully submerged in glass dishes and a ruler for scale. We anesthetized the salp specimens using 0.2% MS-222 prior to photographing them in order to avoid swimming motion in the dishes. We coded the colony architecture for each species we encountered in the field based on our photographs and observations. In addition, we complemented these observations with published records such as Madin (1990) for species we did not encounter, such as *Pegea confoederata, Pegea bicaudata, Thalia democratica, Thalia orientalis, Thetys vagina, Cyclosalpa floridiana, S. younti*, and *Salpa thompsoni*. In addition to categorizing each architecture as transversal, helical, whorl, cluster, oblique, linear, or bipinnate; we also measured the dorsoventral zooid-stolon (zooid oral-aboral axis to stolon axis) angle. To do so, we photographed salp colonies from the dorsoventral-normal (normal *sensu* perpendicular) homologous plane of observation defined in Damian-Serrano & Sutherland (2023a). Using these photographs, we measured the zooid-stolon angle in ImageJ using the aligned endostyle and gill bar as a proxy for the zooid oral-aboral angle, and the line connecting the opaque guts of serially neighboring zooids as a proxy for the stolon angle. For species presenting cluster, whorl, and bipinnate architectures, we used the endostyle as a proxy for the ventral parallel of the zooid axis. We measured at least three zooids per colony (except for *Salpa fusiformis* where we could only measure two zooids) and between one and four individual colonies per species (see SM Table 2). For *Pegea socia*, we used a photograph taken from a specimen collected off the coast of Newport (Oregon, USA) in February 2022. For *S. thompsoni*. we used a frame grab of a video taken from a specimen collected off the coast of Panama in 2005. For *T. democratica*. we used an online photograph by David Shale. For *T. vagina*. we used a frame grab of an online video taken by Patrick Webster off Carmel River (California, USA) in September 2014. For *P. bicaudata*, we used an online photograph taken by Ryu Minemizu off the shore of Kiyan-Cape (Japan) in March 2019.

### Phylogenetic comparative methods

We used the Bayesian time tree to reconstruct the ancestral states of colonial architectures as a categorical character. The developmental ontology in Damian-Serrano & Sutherland (2023a) served as the basis for developing the OMM accounting for the hypothesized homology relationships between architectures, based on the presence or absence of shared developmental mechanisms and pathways. We performed a Bayesian ancestral state reconstruction in RevBayes by constraining the rate matrix to allow only transitions between states that are adjacent in the developmental ontology. To do this, we hard-coded the transition rates between non-adjacent states (e.g., between helical and linear architectures) to be zero, thus requiring states changes across developmental pathways to transition back to a transversal architecture (representing the loss of specific developmental mechanisms) and then shift toward a different pathway following the required order of underlying mechanism gains and losses. This model estimated 12 rate parameters allowing for asymmetrical rates of gain and loss for each transition between architecture states. Alternatively, we repeated this analysis estimating a single rate for all transitions, while still constraining non-adjacent transitions (SM Fig. 4). We used RevBayes for this analysis, adapting the categorical Markov model ancestral state reconstruction protocol described in the “morph_ase” tutorial on the RevBayes website. Alternatively, we reconstructed the ancestral states using stochastic mapping with simpler “equal rates” (single rate parameter for all state transitions with 100 simulations, SM Fig. 5) and “all rates different” (42 independent rate parameters with 25 simulations, one for each rate transition in each direction, SM Fig. 6) models in the R package *phytools* (Revell 2012). While a myriad of alternative models could be estimated and compared, this is beyond the scope of this study. All code scripts and data used for these analyses are available in the Dryad repository (Damian-Serrano & Sutherland 2023b).

While colonial architecture can be conceptualized as a categorical trait, some architectures differ from each other across a gradient in a continuous trait that drives their structural differences. The dorsoventral zooid-stolon angle is one such trait, which drives the streamlining of salp chains. This continuous trait ranges from one extreme with zooids arranged perpendicularly (90°) to the stem of the colony (transversal, helical, whorl, and cluster architectures), all the way to linear chains with zooids arranged in parallel to the stem (as in the linear chains of *Soestia zonaria*), with a gradation of more or less oblique intermediate forms. We reconstructed the evolutionary history of the dorsoventral zooid-stolon angle of salp colonies on the Bayesian time tree using zooid-stolon angle tip values measured from our photographs, and a Brownian Motion model in the R package *phytools* (Revell 2012). We used an ML ancestral state reconstruction with 95% confidence intervals in the R package *ape* 5.6 (Paradis & Schliep 2019). To test the sensitivity of our comparative analyses to phylogenetic uncertainty, we took the 3001 trees generated by the Bayesian topology inference, pruned them to remove the same tips as in the main time tree, and made them ultrametric using R *ape:: chronos*. We ran our continuous estimates of phylogenetic signals on these trees to evaluate the effect of topological uncertainty.

We matched the sequences that form the tips of the molecular phylogeny to the species of the specimens from which we took the morphological data. In the case of *Pegea*, the species we observe off Hawaii has intermediate traits between *P. confoederata* (blastozooid morphology) and *P. socia* (oozooid morphology), possibly representing either a new species, a hybrid, or phenotypic variation within either species. Since we are confident that this is a *Pegea* species and that the genus *Pegea* is likely monophyletic, the branch length for this specimen should be congruent to that of any other *Pegea* species as long as it is the only *Pegea* species in the tree. Thus, we mapped the morphological data of these *Pegea* specimens to the species tip of *P. confoederata* on the phylogeny.

## Results

### Phylogenetic relationships

**Fig. 4.**
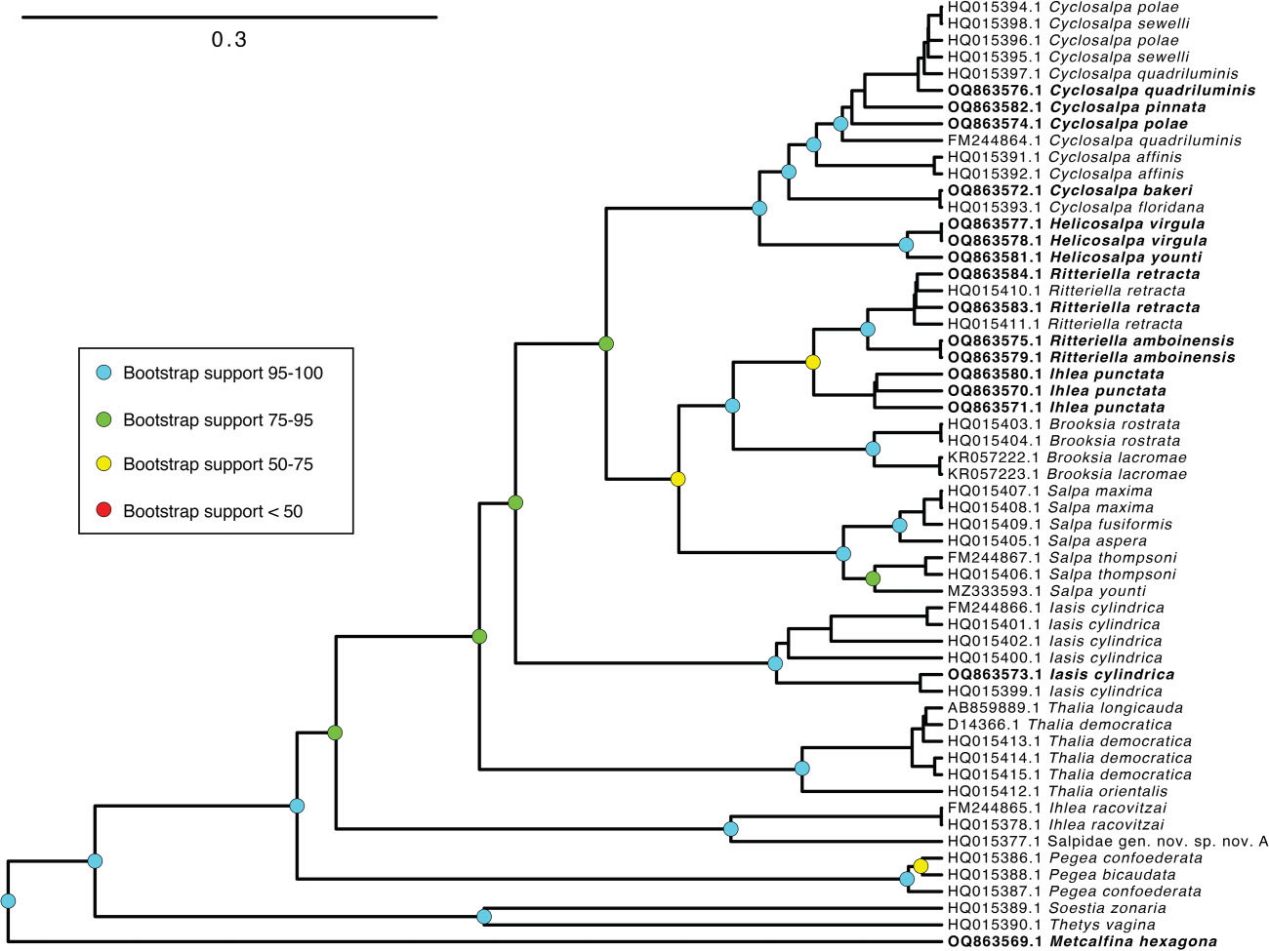
Ultrametric Bayesian time tree inferred from 18S sequences in RevBayes and constrained to be congruent with the ML phylogeny in SM Fig. 1. Branch lengths are estimated using a relaxed molecular clock. Bolded tips correspond to new sequences produced in this study. Outgroups have been removed to facilitate visualization of salp relationships. Bootstrap support of important intermediate and deep nodes indicated with colored circles. Scale bar for phylogenetic distance expressed in nucleotide substitution units.

The phylogenetic relationships between salp species as estimated by ML and Bayesian approaches (SM Figs. 1 and 2) were congruent, except relationships between the *Cyclosalpa* species *C. pinnata, Cyclosalpa polae, Cyclosalpa quadriluminis*, and *Cyclosalpa sewelli* which were unresolved as a polytomy. Our trees reveal for the first time (and with strong support) the position of the genera *Helicosalpa* (*H. virgula and H. younti*) and *Metcalfina* (*M. hexagona*), as well as the species *C. pinnata, C. bakeri, I. punctata*, and *R. amboinensis* in the salp phylogeny (Figs. 3 and 4). We report six novel phylogenetic relationships for salps, including: (1) that the linear-chained *M. hexagona* is sister to all other salp species; (2) that the genus *Helicosalpa* is monophyletic and sister to the genus *Cyclosalpa*; (3) that the genus *Ihlea* as currently described is polyphyletic, with *I. punctata* being sister to the genus *Ritteriella* and not to *Ihlea racovitzai*; (4) that *C. pinnata* is nested within a clade of genetically and morphologically similar *Cyclosalpa* species including *C. polae, C. sewelli*, and *C. quadriluminis*; (5) *that C. bakeri is sister to C. floridiana*; and (6), that *R. amboinensis* is sister to *Ritteriella retracta*. While the node that links *I. punctata* to *Ritteriella* spp. appears unstable (BS 63, PP 0.75), none of the alternative bootstrap topologies place both *Ihlea* species together as a monophyletic clade. As previous studies have found (Tsagkogeorga et al. 2009; Govindarajan et al. 2011), we did not recover the Salpinae as monophyletic, since Cyclosalpidae is nested among them.

Contrary to traditional views, *Pegea* is not the most distant relative to other salps nor is closely related to the *Thetys-Soestia* clade as it appears in the partially supported nodes in Govindarajan et al. (2011). *Pegea* appears to be sister to the clade containing all other salps excluding *Thetys, Soestia*, and *Metcalfina* (BS 79, PP 0.6). In addition, *Thalia* is not the closest relative of *Iasis cylindrica (*formerly known as *Weelia cylindrica*) as indicated in the partially supported node (PP 0.71) in Govindarajan et al. (2011), but instead, it appears to be sister to the clade containing all other salps excluding *I. racovitzai, Pegea, Thetys, Soestia*, and *Metcalfina* (BS 84, PP 0.69). These phylogenetic findings may have important implications for the evolutionary history of salp colony architecture.

The main alternative topologies found among bootstrap replicates comprise alternations of relationships (1) within *Cyclosalpa* species with low support; (2) with the shifting position of *I. racovitzai* as sister to *Pegea*, or as an outgroup to the clade containing *Pegea* and *Salpa*; (3) alternative relationships where *Thalia* is sister to *I. cylindrica*, or appears as a closer relative of the clade containing *Salpa* and *Cyclosalpa* than *I. cylindrica;* and (4) with the shifting position of *I. punctata* as sister to *Brooksia* instead of *Ritteriella*. Since both *Brooksia* and *Ritteriella* have a bipinnate colony architecture and are sister taxa to each other, we predict that the marginal topological uncertainty around these variants has little to no impact on the evolutionary history of colonial architecture in salps, and therefore it was not incorporated into the main analyses.

### Evolutionary history of salp colony architecture

**Fig. 5.**
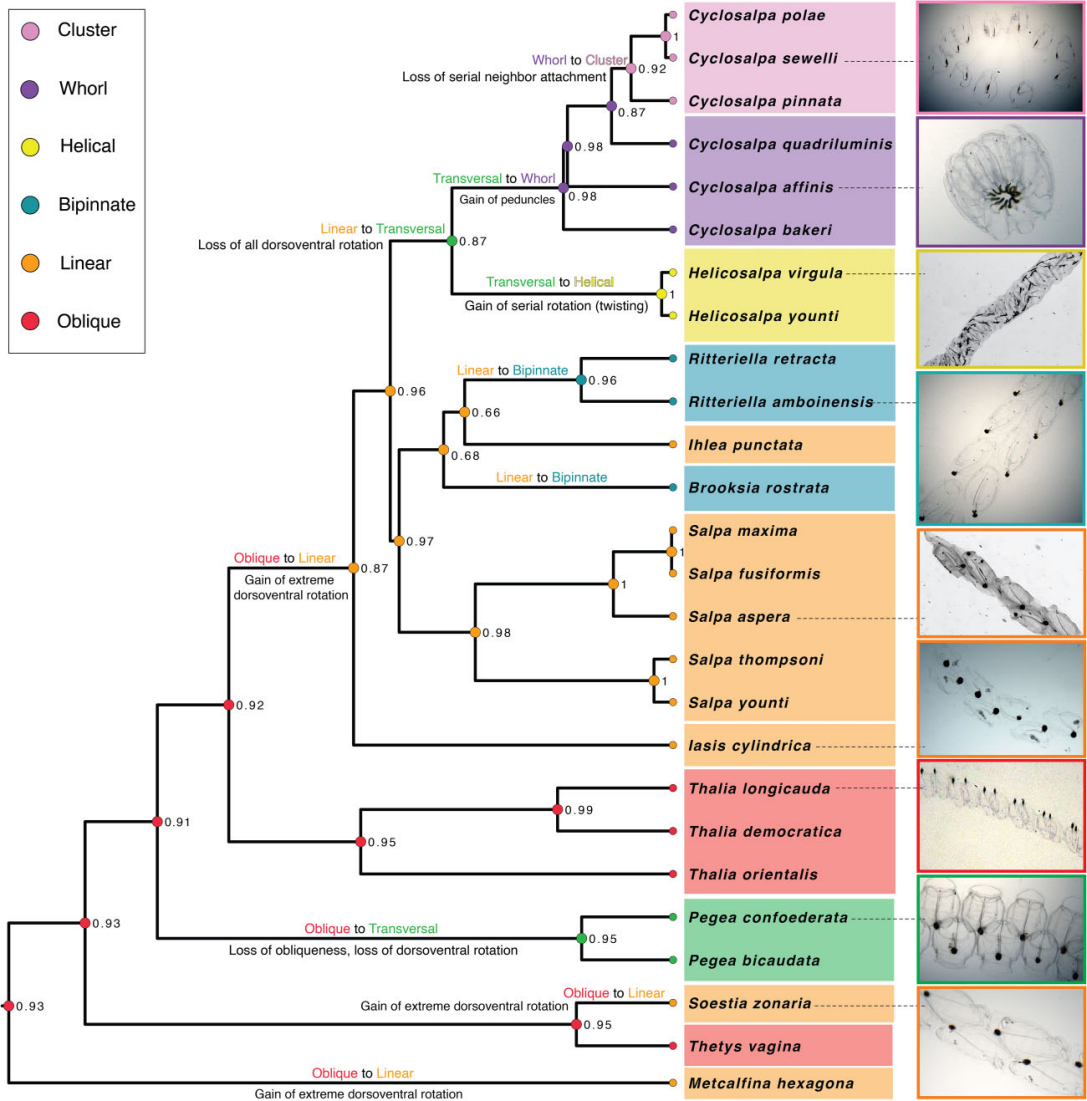
Bayesian ultrametric time tree pruned to display only one representative of each salp species. Species tips are labeled with colored circles indicating their observed colonial architecture. Some species are labeled with an *in situ* photograph derived from brightfield video (Colin et al. 2022) and other *in situ* imaging techniques. Nodes are also labeled with colored circles indicating the most likely ancestral state and their PPs. Ancestral states and their PPs were computed with a Bayesian OMM model in RevBayes using a custom rate matrix with 12 rate parameters constrained to the developmental transition pathways detailed in Damian-Serrano & Sutherland (2023a). State transitions are labeled and described on the branches where they occur.

We used a Bayesian ancestral state reconstruction analysis of salp colony architecture (coded as a categorical character) using an OMM matrix to reveal the evolutionary history of this complex trait (Fig. 5). We find that the most likely state of the most recent common ancestor of salps is the oblique chain architecture (PP 0.95), with a marginal PP (0.04) of being linear. This ancestral oblique architecture is then retained in *Thetys* and *Thalia*. From this ancestral state, we observe the independent evolution of the linear colony architecture in three lineages including *M. hexagona, S. zonaria*, and the ancestor of the clade containing *Salpa* and *Cyclosalpa*. This linear architecture is then retained in the genera *Salpa* and *Iasis*. A fourth independent evolution of linear architecture is partially supported (PP 0.34) in *I. punctata* in the scenario where its common ancestor with *Brooksia rostrata* is reconstructed as bipinnate, followed by the secondary loss of the bipinnate architecture back to a linear form in *I. punctata*. Thus, the bipinnate architecture has either evolved twice independently (in *B. rostrata* and in *Ritteriella* spp., with a PP of 0.68) or once (PP 0.32) and then lost in *I. punctata*.

The dorsoventral zooid rotation mechanism that gives rise to oblique and linear chains is lost twice independently, once in the lineage leading to *Pegea* spp. (PP 0.95), and again in the lineage leading to the ancestor of *Cyclosalpa* and *Helicosalpa*. From this latter hypothetically transversal-chained ancestor, the helical architecture evolved once in the lineage leading to the genus *Helicosalpa* with the gain of stolon twisting. On the other hand, its sister lineage evolved the whorl architecture via the continuous growth mode of developing blastozooids and the development of peduncles, leading to the common ancestor of the genus *Cyclosalpa* (PP 0.98). Several *Cyclosalpa* species (*Cyclosalpa affinis, C. bakeri*, and *C. quadriluminis*) retain this whorl architecture, while the common ancestor of the subclade containing *C. polae, C. sewelli*, and *C. pinnata* evolved from a whorl architecture to a cluster form (PP 0.92) through the loss of attachment between serial neighbors.

The Bayesian OMM reconstruction with a single rate (SM Fig. 3) was congruent with the 12-rate reconstruction, with even stronger PP support of the reconstructed ancestral states across nodes. We also reconstructed this evolutionary history using stochastic mapping (SIMMAP) with alternative simpler models such as “equal rates” (SM Fig. 4) and “all rates different” (SM Fig. 5). These reconstructions are mostly congruent with the OMM reconstruction in Fig. 3, but present great uncertainty on the ancestral states of deep nodes of the phylogeny. The “equal rates” model reconstructs the common ancestor of *Helicosalpa* and *Cyclosalpa* as a whorl architecture, while the “all rates different” model reconstructs it as a cluster architecture. Both models had slightly stronger support for a linear state at the MRCA retained along most lineages with two independent gains of oblique colony architecture.

**Fig. 6.**
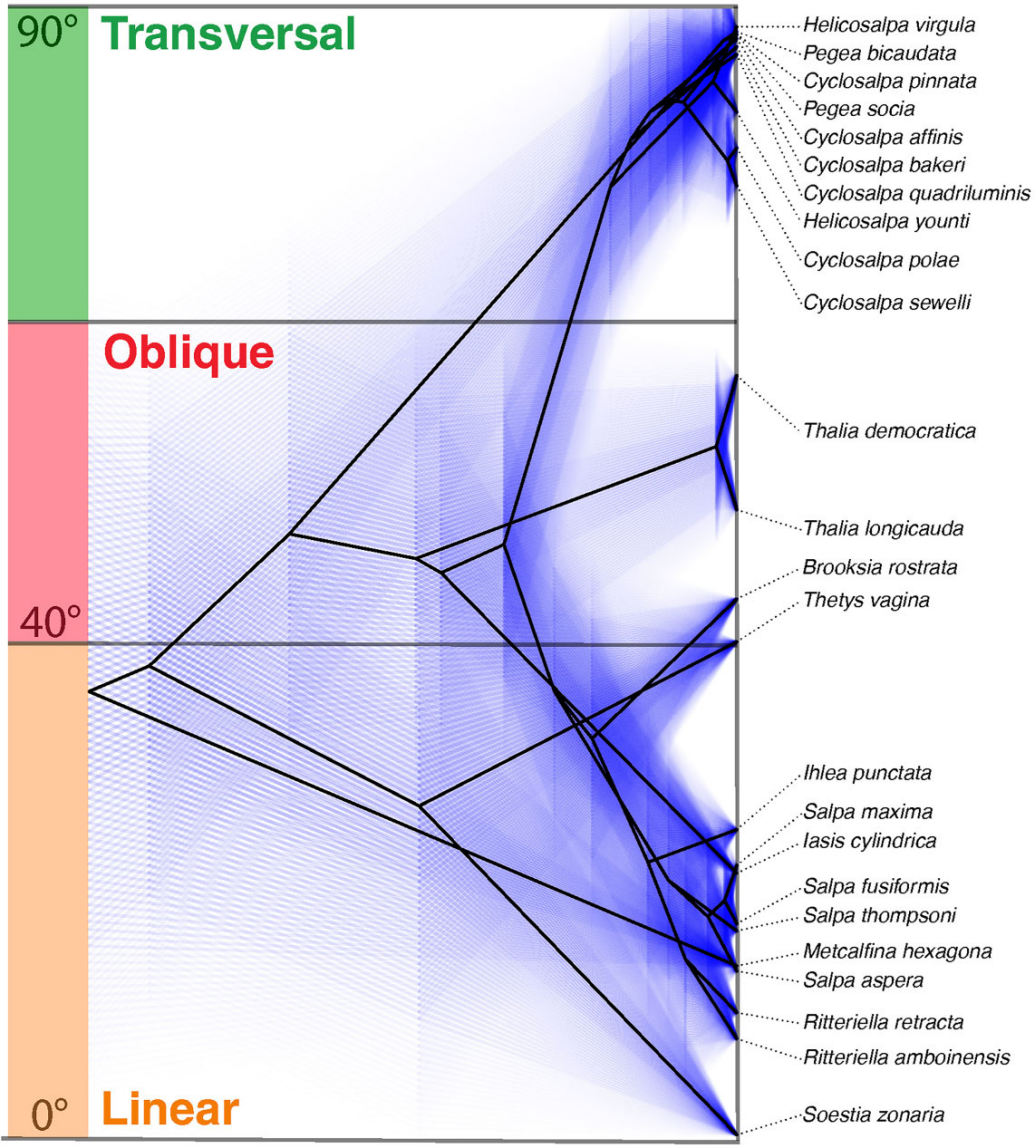
Continuous reconstruction of dorsoventral zooid-stolon angle evolution with BM. Black lines indicate the average ancestral states, while faint blue lines represent the 95% confidence intervals from an ML ancestral state reconstruction.

We calculated the phylogenetic signal of the dorsoventral zooid-stolon angle across salp species using Blomberg's K (Blomberg et al. 2003) with the species’ means and standard errors (accounting for intraspecific variation) and obtained a significant and strong signal (K of 1.61, *P*-value of 0.001), indicating phylogenetic conservatism (K > 1) of this trait. This signal ranged between 0.97 and 1.9 across all bootstrap tree topologies (1st quartile = 1.42, 3rd quartile = 1.65), indicating it is robust to phylogenetic uncertainty. We then reconstructed the evolutionary history of the species' mean zooid-stolon angle under a single-rate Brownian Motion (BM) model (Fig. 6). This reconstruction shows that the MRCA of all salps was likely either linear or oblique, with two independent losses of dorsoventral zooid-stolon rotation in *Pegea* and Cyclosalpidae (*Cyclosalpa* and *Helicosalpa*) respectively, in agreement with the categorical reconstruction. Moreover, this continuous trait approach shows that dorsoventral zooid-stolon angles in the oblique range evolved thrice, with the bipinnate *B. rostrata* presenting wider angles than *T. vagina* (a species traditionally considered oblique), the latter presenting a 40.49° angle that approaches the 40° threshold of linearity. Finally, this reconstruction also shows that linearity has evolved at least three (assuming a linear MRCA) or four (assuming an oblique MRCA) times independently, in agreement with the findings from the categorical OMM reconstruction.

## Discussion

Salp colonies present a striking diversity of three-dimensional architectures that is unique among colonial organisms. The evolutionary history of these architectures has eluded scientists for decades due to a lack of a comparative characterization of architectural variation and a lack of phylogenetic taxon sampling. Here we present a phylogenetic tree for salps including several novel sequences that have led to the resolution of formerly ambiguous nodes, as well as the discovery of the phylogenetic relationships of understudied yet critical lineages of salps. In addition, we leveraged this phylogeny as well as a recently published developmental ontology of colonial architectures and new measurements of zooid orientations to reconstruct the elusive evolutionary history of the striking architectural diversity among salp colonies.

In the absence of reliable means to empirically reconstruct this evolutionary history, researchers have generated some hypotheses based on colonial development and complexity. Transversal architectures are developmentally basal as the initial primordial state of newly budded blastozooids (Damian-Serrano & Sutherland 2023a). This developmentally simple state was hypothesized to be the ancestral state of colonial architecture in the MRCA of salps (Madin 1974). Therefore, species that retain a transversal architecture through adulthood have been hypothesized to be the sister taxon of all other salps (Madin 1990), where species with more developmentally derived architectures (such as linear chains) shared more recent common ancestry and such architectures as a synapomorphy. Moreover, species with colonial architectures that lack dorsoventral zooid-stolon rotation such as whorls and clusters were hypothesized to be closer relatives of those with transversal architectures (Madin 1974; Govindarajan et al. 2011).

Our results show that the salp sister taxon to all other salps (*M. hexagona*) presents a linear architecture and that transversal forms like *Pegea* are nested among oblique and linear forms. Moreover, we find that transversal-like forms (helical chains, whorls, and clusters) are not closely related to transversal forms and have evolved independently. Our ancestral reconstructions using both continuous and categorical characters further support that the developmental invariance in the colony architecture of *Pegea* is derived, not ancestral, marked by a loss of ancestral zooid rotation mechanisms. Our findings overturn the hypothesis that transversal forms are less derived but cast a complex picture of the evolutionary history of linearity. While the dorsoventral zooid-stolon rotation mechanism (shared by oblique and transversal forms) responsible for linearity is present in the MRCA, accentuations in the degree of linearity (from likely oblique ancestors) have been gained several times independently, partially supporting the hypothesis that linear forms are derived. These findings profoundly change the paradigm of salp evolution. These findings are contingent on the phylogenetic position of *Thalia* and *Pegea* in the tree. While the support values in our tree are moderately robust, further efforts in phylogenetic reconstruction using larger genomic datasets may reveal alternate topologies that could alter these conclusions.

In Damian-Serrano & Sutherland (2023a), the bipinnate architecture is described as a derived state following the development of an intermediary linear architecture. This is congruent with the dorsoventral zooid-stolon angle measurements we recorded for *Ritteriella* spp. However, our quantitative measurements for this angle for *Brooksia* show that while this taxon does present dorsoventral zooid rotation in its early development, it does not undergo a full transition to linear before rotating and flaring its zooids into a bipinnate position. This represents an exception to the ontology that suggests that the developmental mechanisms that give rise to each architecture operate independently of each other. As more salp diversity is uncovered and characterized, we expect to find more of these novel combinations of architectural traits.

We hypothesize that the simpler “equal rates” and “all rates different” models fail to reconstruct realistic ancestral states since they assume it is equally likely to transition between developmentally adjacent states (such as oblique to linear) as it is to transition from one terminally derived architecture to another (such as linear to cluster), without requiring intermediate steps of gain and loss of dorsoventral zooid-stolon rotation or asynchronous zooid development with peduncles. These developmental mechanisms can also be modeled as independent characters, which we hypothesize would yield very similar results as our ordered rate matrix reconstruction. This is because modifying the aggregation of characters is equivalent to modifying the aggregation of rates (Tarasov 2019). Furthermore, while we aggregated all linear colonial architectures as the same (hypothetically homologous) state, we hypothesize that the multiple independent origins of this architecture may have different developmental underpinnings. Further research comparing the detailed anatomy of zooid-zooid connections across *Metcalfina, Soestia, Iasis, Ihlea* and *Salpa* colonies may be able to assess their homology.

Since the true model of evolution is unknown, we considered an alternative continuous model in addition to a categorical ordered Markov process and an equal rates stochastic mapping. To explore a gradual evolution approach, we used a BM model that would capture the gradual variation in zooid angles observed among species with oblique and linear chains. This model was congruent with the ancestral reconstruction conclusions drawn from the categorical approach. While it was unable to distinguish between transversal, helical, whorl, and cluster forms; it did provide more detailed insights into the phylogenetic distribution of “linearity” across oblique, bipinnate, and linear colonies. For example, this continuous comparative analysis revealed that some bipinnate chains (such as *B. rostrata*) are in fact less linear than traditionally oblique species (such as *T. vagina*).

The evolutionary history of salp colony architecture generates hypotheses on the functionality of the different colonial forms. Salp colonies move in the water column as a single animal through coordinated multi-jet propulsion that emerges from the sum of pulsatile jets of each zooid's excurrent siphon (Sutherland & Weihs 2017). The differential arrangement of blastozooids in a colony will likely affect the orientation of the propulsive jets to each other and to the overall colony motion axis. In Damian-Serrano & Sutherland (2023a), we hypothesized that different architectures would differ in how cross-sectional area scales with the number and size of propeller zooids, as a function of its motion-orthogonal frontal drag. Moreover, we hypothesized that the angle of excurrent jets relative to the motion axis will depend on colony architecture and impact the thrust-to-torque ratio. These hydrodynamic properties may determine the propulsive efficiency of different architectures. Linear chains are hypothesized to present the most efficient hydrodynamic properties (Bone & Trueman 1983). Natural selection may favor architectural variants with greater propulsive efficiency in response to pressures such as predation, habitat patchiness, and vertical migration behavior. Our results suggest that linear chain architecture has re-evolved several times independently, more often than any other architecture. This is congruent with a scenario where linear architecture is favored across multiple ecological contexts. However, our results also indicate that linear architectures (or near-linear oblique architectures) may be ancestral, indicating that the set of traits required for high locomotory performance was lost multiple times with the evolution of transversal, helical, whorl, and cluster forms. Many of these species are not long-distance vertical migrators (Madin et al. 1996), which may lead to reduced selective pressure on hydrodynamic efficiency, allowing for the evolution of alternative configurations of zooids. However, the ecological advantages conferred by these other architectures (if any) remain unknown.

In the past decade, salps have attracted the attention of oceanographers given their role as consumers of microbial and primary production in pelagic ecosystems (Henschke et al. 2016). Salp fecal pellets are responsible for a large fraction of the biological carbon pump (Décima et al. 2023) that exports large quantities of phytoplankton-fixed carbon fixed into deep waters. However, most studies remain focused on a few species (typically within *Salpa* and *Thalia*) while the bulk of salp biodiversity remains understudied. This phylogeny will help biologists contextualize knowledge from different salp species from an evolutionary perspective.

## Supplementary Material

obad037_Supplemental_Files

## Data Availability

Data and code for this study are available on our Dryad repository: Damian-Serrano & Sutherland (2023b).

## References

[bib1] Blomberg SP , Garland T Jr, Ives AR. 2003. Testing for phylogenetic signal in comparative data: behavioral traits are more labile. Evolution 57:717–45. 12778543 10.1111/j.0014-3820.2003.tb00285.x

[bib2] Bone Q . 1998. The Biology of Pelagic Tunicates. Oxford: Oxford University Press.

[bib3] Bone Q , Trueman ER. 1983. Jet propulsion in salps (Tunicata: thaliacea). J Zool 201:481–506.

[bib4] Castresana J . 2000. Selection of conserved blocks from multiple alignments for their use in phylogenetic analysis. Mol Biol Evol 17:540–52. 10742046 10.1093/oxfordjournals.molbev.a026334

[bib5] Colin SP , Gemmell BJ, Costello JH, Sutherland KR. 2022 In situ high-speed brightfield imaging for studies of aquatic organisms v.2. protocols.io. 10.17504/protocols.io.kxygxz4ykv8j/v2

[bib6] Damian-Serrano A , Hetherington ED, Choy CA, Haddock SH, Lapides A, Dunn CW. 2022. Characterizing the secret diets of siphonophores (Cnidaria: hydrozoa) using DNA metabarcoding. PLoS One 17:e0267761. 35594271 10.1371/journal.pone.0267761 PMC9122208

[bib7] Damian-Serrano A , Sutherland KR. 2023a. A developmental ontology for the colonial architecture of salps. *BioRxiv* 10.1101/2023.09.04.555288 38820292

[bib8] Damian-Serrano A , Sutherland KR. 2023b, A new molecular phylogeny of salps (Tunicata: thalicea: salpida) and the evolutionary history of their colonial architecture. Dryad, Dataset, 10.5061/dryad.3r2280gn1 PMC10576244 37840689

[bib9] Décima M , Stukel MR, Nodder SD, Gutiérrez-Rodríguez A, Selph KE, Dos Santos AL, … Pinkerton M. 2023. Salp blooms drive strong increases in passive carbon export in the Southern Ocean. Nat Commun 14:425. 36732522 10.1038/s41467-022-35204-6 PMC9894854

[bib10] Dunn CW , Hejnol A, Matus DQ, Pang K, Browne WE, Smith SA, Giribet G. 2008. Broad phylogenomic sampling improves resolution of the animal tree of life. Nature 452:745–9. 18322464 10.1038/nature06614

[bib11] Edgar RC . 2004. MUSCLE: a multiple sequence alignment method with reduced time and space complexity. BMC Bioinf 5:1–19. 10.1186/1471-2105-5-113 PMC517706 15318951

[bib12] Govindarajan AF , Bucklin A, Madin LP. 2011. A molecular phylogeny of the Thaliacea. J Plankton Res 33:843–53.

[bib13] Haddock SH , Heine JN. 2005. Scientific blue-water diving, vol. 1, La Jolla, CA: California Sea Grant College Program.

[bib14a] Henschke N , Everett JD, Richardson AJ, Suthers IM. 2016. Rethinking the role of salps in the ocean. Trends in ecology & evolution 31(9):720–33. 27444105 10.1016/j.tree.2016.06.007

[bib14] Höhna S , Landis MJ, Heath TA, Boussau B, Lartillot N, Moore BR, Ronquist F. 2016. RevBayes: bayesian phylogenetic inference using graphical models and an interactive model-specification language. Syst Biol 65:726–36. 27235697 10.1093/sysbio/syw021 PMC4911942

[bib15] Katoh K , Asimenos G, Toh H. 2009. Multiple alignment of DNA sequences with MAFFT. Methods Mol Biol 65:39–64. 10.1007/978-1-59745-251-9_3 19378139

[bib16] Madin LP . 1974. Field Studies on the Biology of Salps (Tunicata: Thaliacea). California: University of California, Davis.

[bib17] Madin LP . 1990. Aspects of jet propulsion in salps. Can J Zool 68:765–77.

[bib18] Madin LP , Kremer P, Hacker S. 1996. Distribution and vertical migration of salps (Tunicata, Thaliacea) near Bermuda. J Plankton Res 18:747–55.

[bib19] Nguyen LT , Schmidt HA, Von Haeseler A, Minh BQ. 2015. IQ-TREE: a fast and effective stochastic algorithm for estimating maximum-likelihood phylogenies. Mol Biol Evol 32:268–74. 25371430 10.1093/molbev/msu300 PMC4271533

[bib20] Paradis E , Schliep K. 2019. ape 5.0: an environment for modern phylogenetics and evolutionary analyses in R. Bioinformatics 35:526–8. 30016406 10.1093/bioinformatics/bty633

[bib21] Revell LJ . 2012. phytools: an R package for phylogenetic comparative biology (and other things). Methods Ecol Evol 3:217–23.

[bib22] Sutherland KR , Weihs D. 2017. Hydrodynamic advantages of swimming by salp chains. J R Soc, Interface 14: 20170298. 28768881 10.1098/rsif.2017.0298 PMC5582125

[bib23] Tarasov S . 2019. Integration of anatomy ontologies and evo-devo using structured Markov models suggests a new framework for modeling discrete phenotypic traits. Syst Biol 68:698–716. 30668800 10.1093/sysbio/syz005 PMC6701457

[bib24] Tsagkogeorga G , Turon X, Hopcroft RR, Tilak MK, Feldstein T, Shenkar N, … Delsuc F. 2009. An updated 18S rRNA phylogeny of tunicates based on mixture and secondary structure models. BMC Evol Biol 9:1–16. 19656395 10.1186/1471-2148-9-187 PMC2739199

[bib25] Wagner GP , Chiu CH, Laubichler M. 2000. Developmental evolution as a mechanistic science: the inference from developmental mechanisms to evolutionary processes. Am Zool 40:819–31.

