# Supplementary material for: A New Molecular Phylogeny of Salps (Tunicata: Thalicea: Salpida) and the Evolutionary History of Their Colonial Architecture
Source: Integr Org Biol. 2023 Sep 27;5(1):obad037. doi: 10.1093/iob/obad037 (PMC10576244; doi:10.1093/iob/obad037)
Supplement: obad037_Supplemental_Files [file obad037_supplemental_files.zip › PhylogenyMS_Supplement_revised.docx]

**Supplementary Materials**


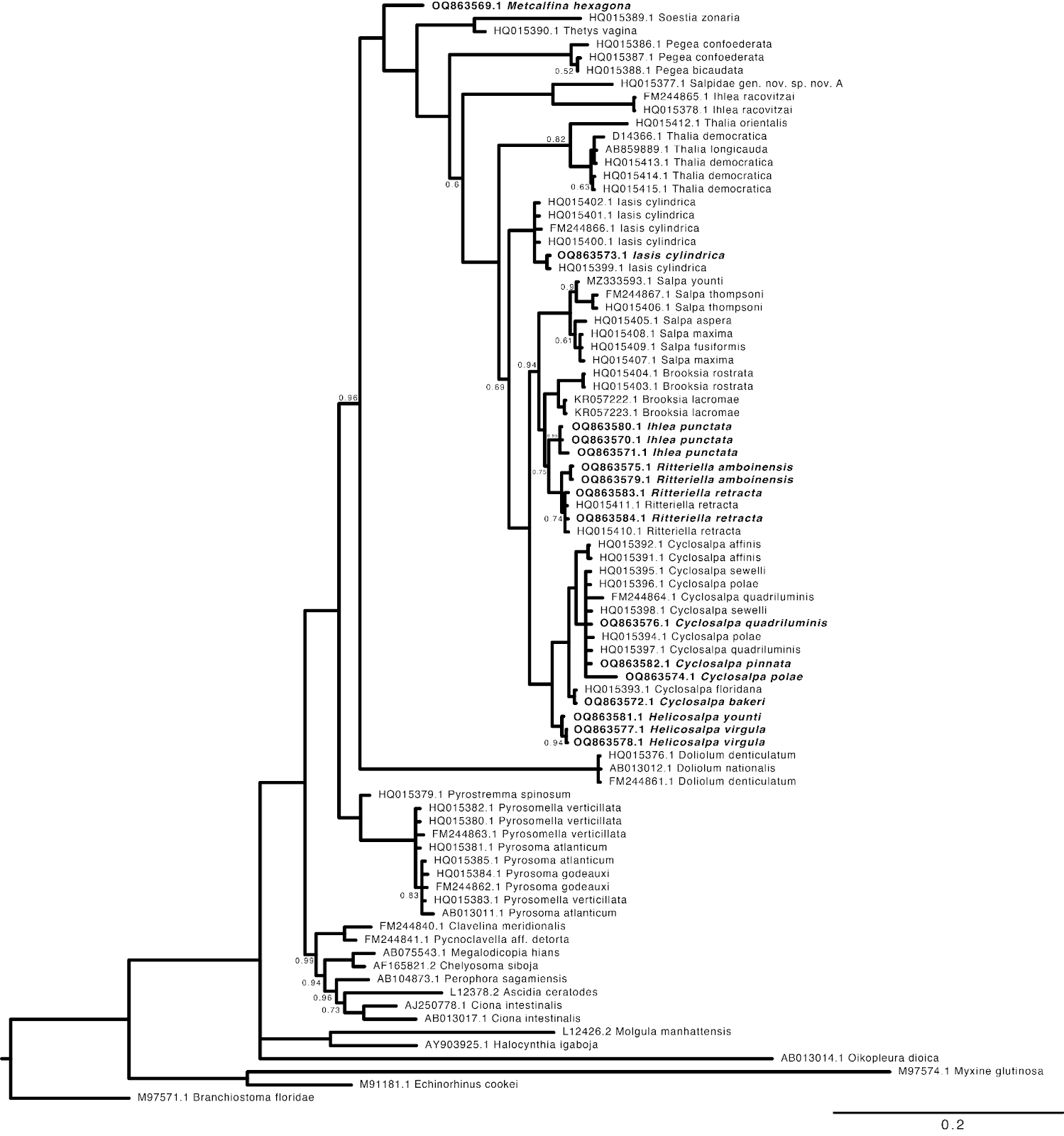
 SM Figure 1. Bayesian phylogeny. Nodes labeled with posterior support. Unlabeled nodes have posterior support of 1. Tip labels in bold font are new additions from this study.


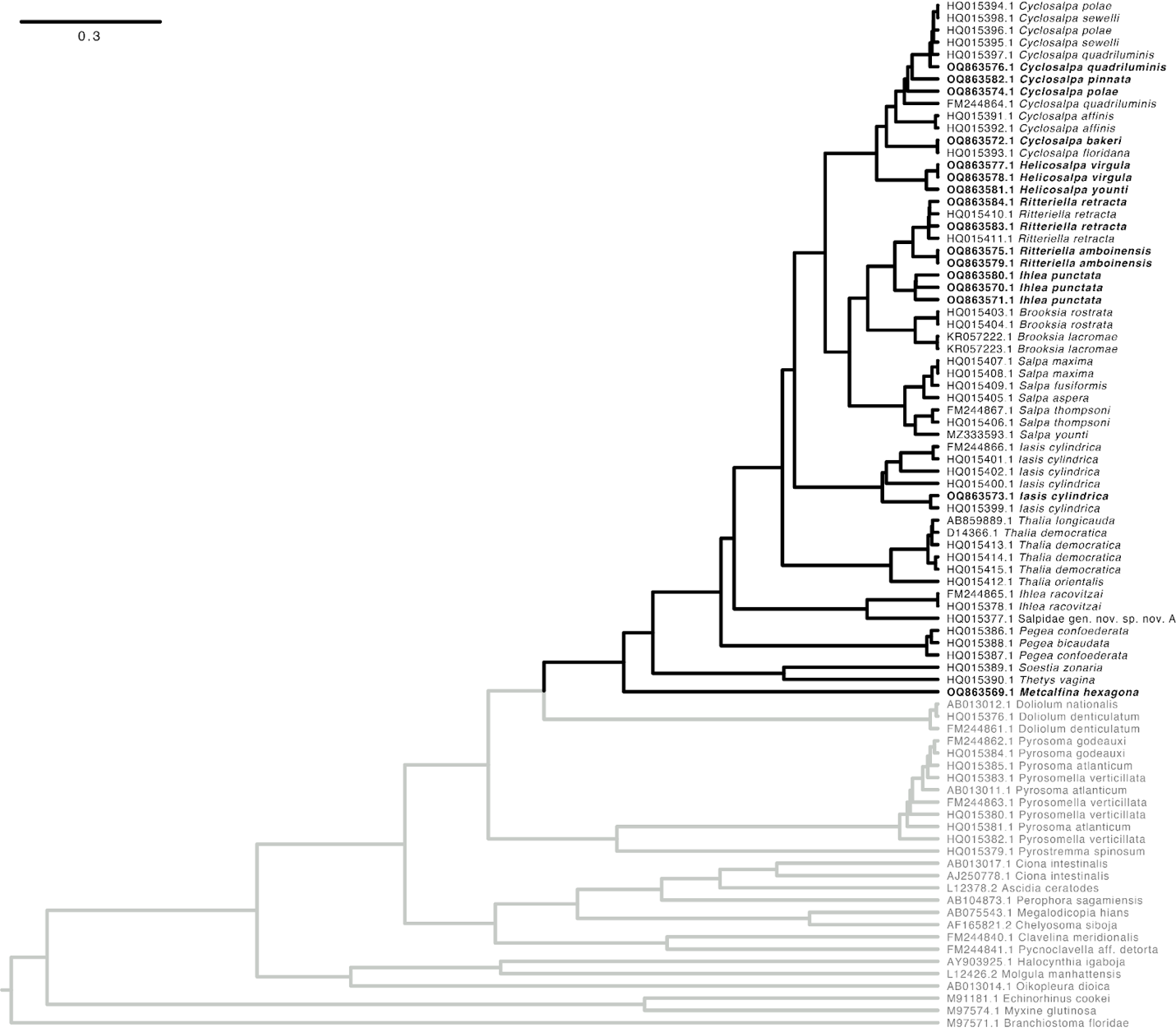


SM Figure 2. Ultrametric Bayesian time tree inferred from 18S sequences in RevBayes and constrained to be congruent with the ML phylogeny in SM Figure 1. Branch lengths are estimated using a relaxed molecular clock. Grayed-out tips and branches correspond to non-salp outgroups used in the inference. Bolded tips correspond to new sequences produced in this study.


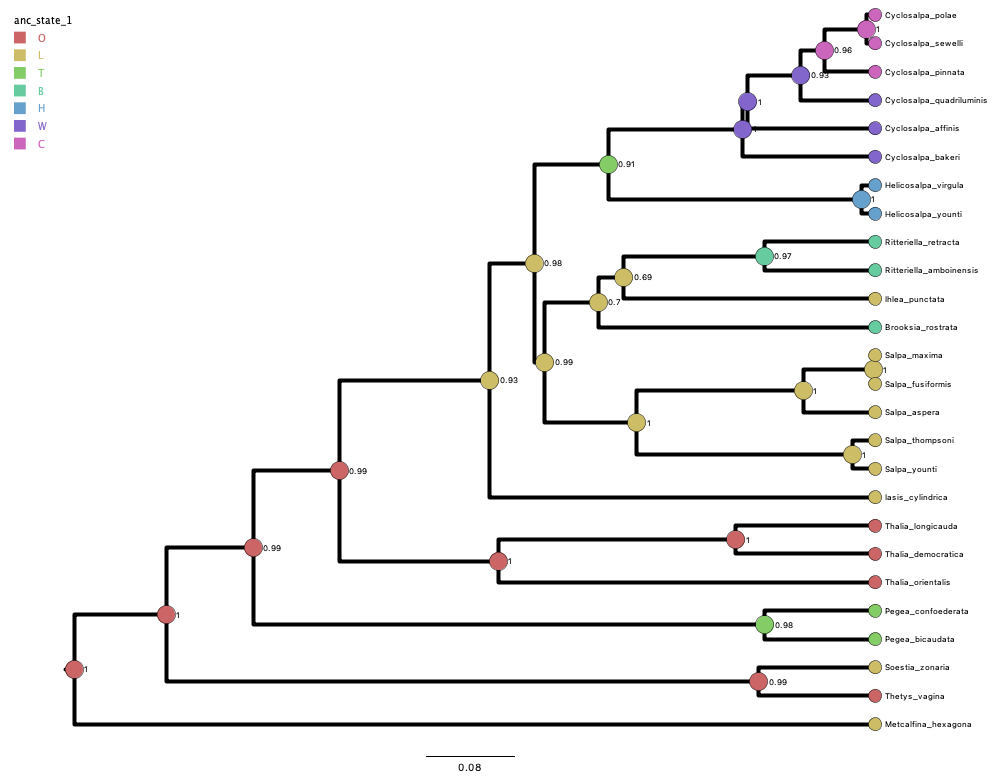


SM Figure 3. Bayesian ultrametric time tree pruned to display only one representative of each salp species. Species tips are labeled with colored circles indicating their observed colonial architecture. Nodes are also labeled with colored circles indicating the most likely ancestral state and their PPs. Ancestral states and their PPs were computed with a Bayesian OMM model in RevBayes using a custom single-rate matrix constrained to the developmental transition pathways detailed in Damian-Serrano & Sutherland (2023).


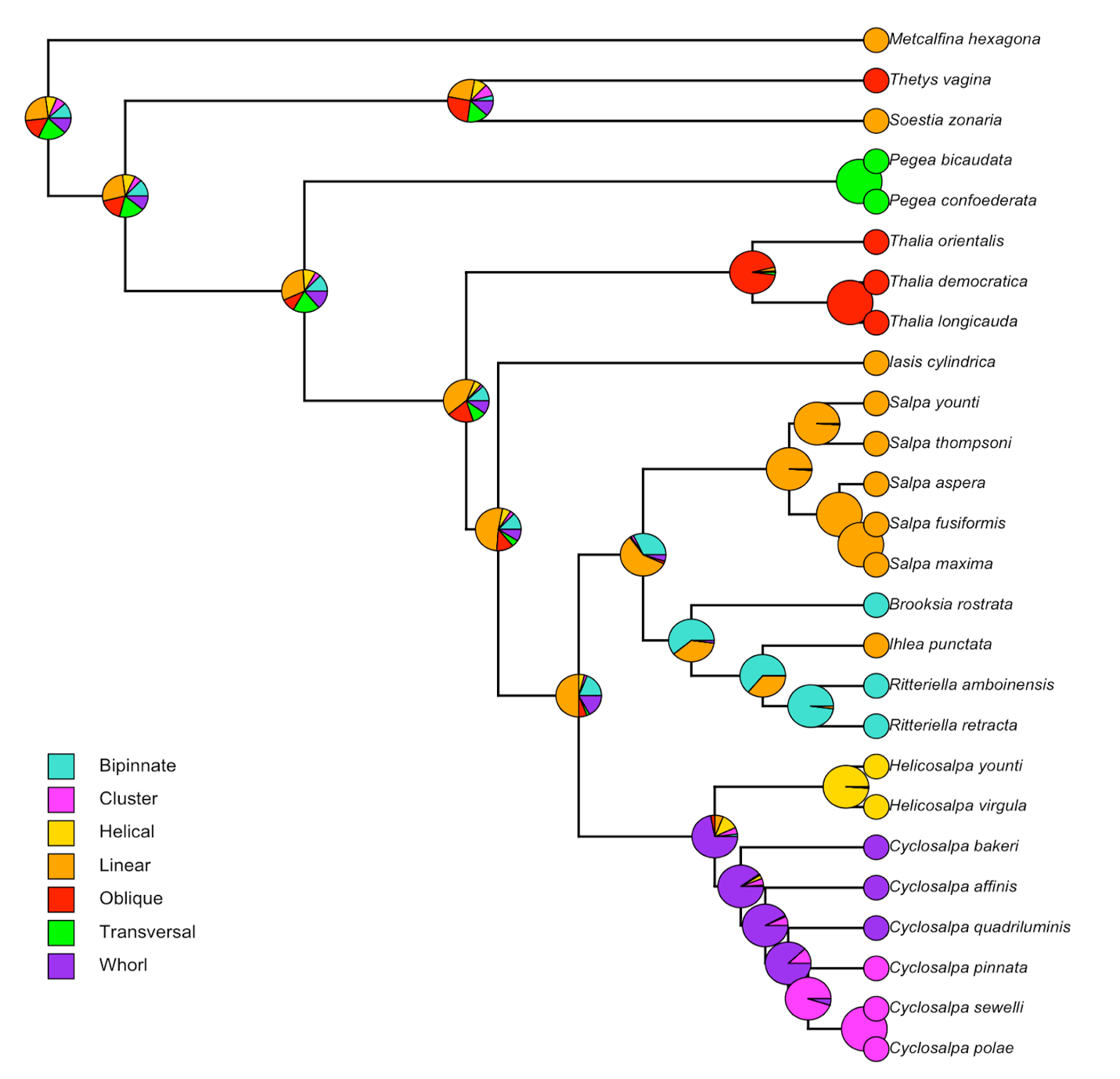


SM. Figure 4. Bayesian ultrametric time tree pruned to display only one representative of each salp species. Species tips are labeled with colored circles indicating their observed colonial architecture. Nodes are labeled with pie charts indicating ancestral state probabilities mapped using an “equal rates” SIMMAP model.


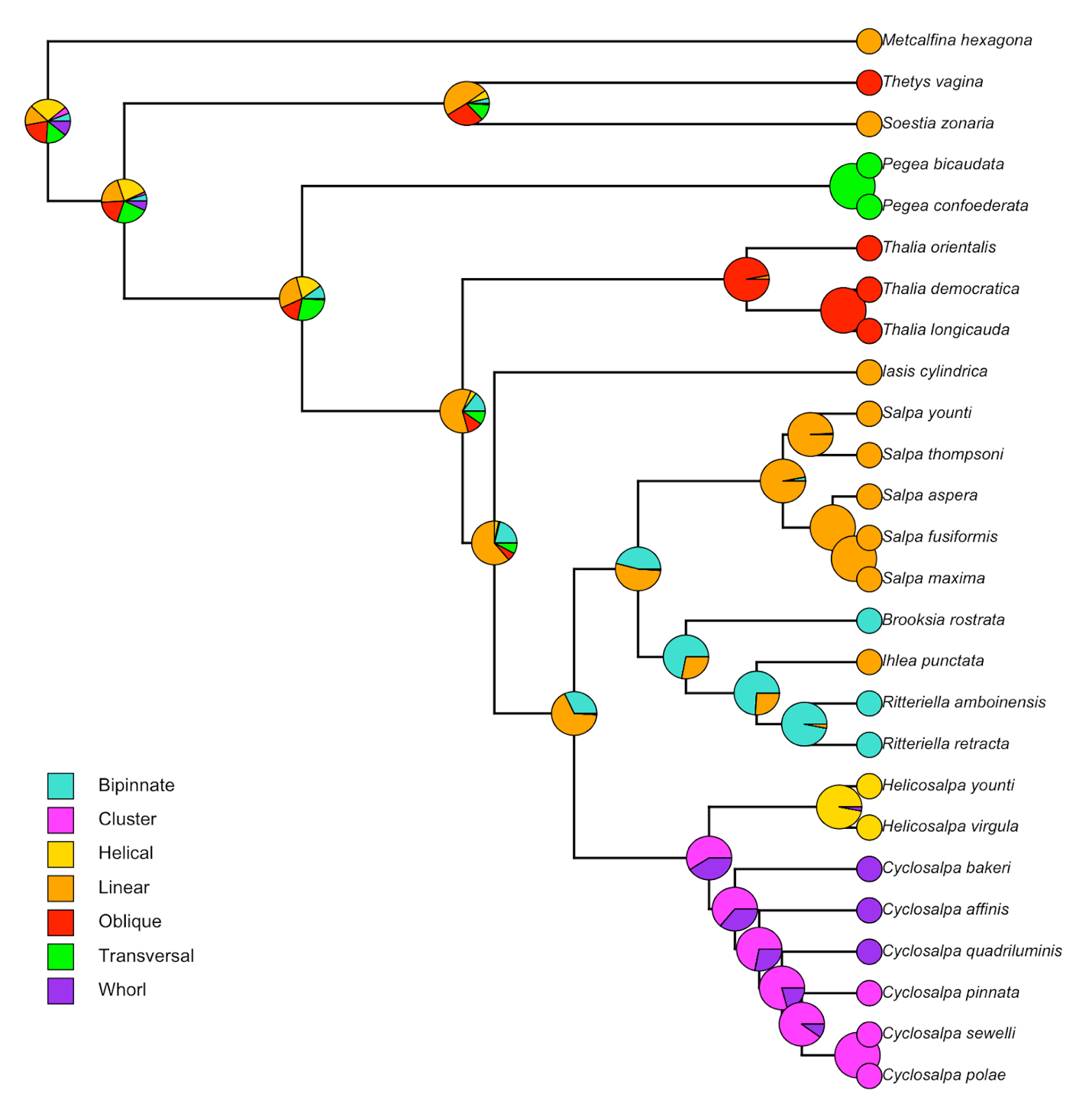


SM. Figure 5. Bayesian ultrametric time tree pruned to display only one representative of each salp species. Species tips are labeled with colored circles indicating their observed colonial architecture. Nodes are labeled with pie charts indicating ancestral state probabilities mapped using an “all rates different” SIMMAP model.

SM Table 1. List of the 18S sequence accessions used in the phylogenetic inference analyses.

| Accession number | Species | Sequence type | Collection Date |
| --- | --- | --- | --- |
| OQ863569.1 | *Metcalfina hexagona* | New salp sequence | 14-Sep-2022 |
| OQ863570.1 | *Ihlea punctata* | New salp sequence | 24-Apr-2022 |
| OQ863571.1 | *Ihlea punctata* | New salp sequence | 24-Apr-2022 |
| OQ863572.1 | *Cyclosalpa bakeri* | New salp sequence | 26-Jun-2022 |
| OQ863573.1 | *Iasis cylindrica* | New salp sequence | 22-Apr-2022 |
| OQ863574.1 | *Cyclosalpa polae* | New salp sequence | 14-Sep-2022 |
| OQ863575.1 | *Ritteriella amboinensis* | New salp sequence | 24-Apr-2022 |
| OQ863576.1 | *Cyclosalpa quadriluminis* | New salp sequence | 14-Sep-2022 |
| OQ863577.1 | *Helicosalpa virgula* | New salp sequence | 16-Sep-2022 |
| OQ863578.1 | *Helicosalpa virgula* | New salp sequence | Apr-2021 |
| OQ863579.1 | *Ritteriella amboinensis* | New salp sequence | 27-Jun-2022 |
| OQ863580.1 | *Ihlea punctata* | New salp sequence | 30-Jun-2022 |
| OQ863581.1 | *Helicosalpa younti* | New salp sequence | 26-Jun-2022 |
| OQ863582.1 | *Cyclosalpa pinnata* | New salp sequence | 27-Jun-2022 |
| OQ863583.1 | *Ritteriella retracta* | New salp sequence | 13-Sep-2022 |
| OQ863584.1 | *Ritteriella retracta* | New salp sequence | 13-Sep-2022 |
| FM244864.1 | *Cyclosalpa quadriluminis* | Salp sequence | Retrieved from GenBank |
| FM244865.1 | *Ihlea racovitzai* | Salp sequence | Retrieved from GenBank |
| FM244866.1 | *Iasis cylindrica* | Salp sequence | Retrieved from GenBank |
| FM244867.1 | *Salpa thompsoni* | Salp sequence | Retrieved from GenBank |
| HQ015377.1 | Salpidae gen. nov. sp. nov. A | Salp sequence | Retrieved from GenBank |
| HQ015406.1 | *Salpa thompsoni* | Salp sequence | Retrieved from GenBank |
| HQ015415.1 | *Thalia democratica* | Salp sequence | Retrieved from GenBank |
| HQ015414.1 | *Thalia democratica* | Salp sequence | Retrieved from GenBank |
| HQ015413.1 | *Thalia democratica* | Salp sequence | Retrieved from GenBank |
| HQ015412.1 | *Thalia orientalis* | Salp sequence | Retrieved from GenBank |
| HQ015411.1 | *Ritteriella retracta* | Salp sequence | Retrieved from GenBank |
| HQ015410.1 | *Ritteriella retracta* | Salp sequence | Retrieved from GenBank |
| HQ015409.1 | *Salpa fusiformis* | Salp sequence | Retrieved from GenBank |
| HQ015408.1 | *Salpa maxima* | Salp sequence | Retrieved from GenBank |
| HQ015407.1 | *Salpa maxima* | Salp sequence | Retrieved from GenBank |
| HQ015405.1 | *Salpa aspera* | Salp sequence | Retrieved from GenBank |
| HQ015404.1 | *Brooksia rostrata* | Salp sequence | Retrieved from GenBank |
| HQ015403.1 | *Brooksia rostrata* | Salp sequence | Retrieved from GenBank |
| HQ015402.1 | *Iasis cylindrica* | Salp sequence | Retrieved from GenBank |
| HQ015401.1 | *Iasis cylindrica* | Salp sequence | Retrieved from GenBank |
| HQ015400.1 | *Iasis cylindrica* | Salp sequence | Retrieved from GenBank |
| HQ015399.1 | *Iasis cylindrica* | Salp sequence | Retrieved from GenBank |
| HQ015398.1 | *Cyclosalpa sewelli* | Salp sequence | Retrieved from GenBank |
| HQ015397.1 | *Cyclosalpa quadriluminis* | Salp sequence | Retrieved from GenBank |
| HQ015396.1 | *Cyclosalpa polae* | Salp sequence | Retrieved from GenBank |
| HQ015395.1 | *Cyclosalpa sewelli* | Salp sequence | Retrieved from GenBank |
| HQ015394.1 | *Cyclosalpa polae* | Salp sequence | Retrieved from GenBank |
| HQ015393.1 | *Cyclosalpa floridana* | Salp sequence | Retrieved from GenBank |
| HQ015392.1 | *Cyclosalpa affinis* | Salp sequence | Retrieved from GenBank |
| HQ015391.1 | *Cyclosalpa affinis* | Salp sequence | Retrieved from GenBank |
| HQ015390.1 | *Thetys vagina* | Salp sequence | Retrieved from GenBank |
| HQ015389.1 | *Soestia zonaria* | Salp sequence | Retrieved from GenBank |
| HQ015388.1 | *Pegea bicaudata* | Salp sequence | Retrieved from GenBank |
| HQ015387.1 | *Pegea confoederata* | Salp sequence | Retrieved from GenBank |
| HQ015386.1 | *Pegea confoederata* | Salp sequence | Retrieved from GenBank |
| HQ015378.1 | *Ihlea racovitzai* | Salp sequence | Retrieved from GenBank |
| KR057223.1 | *Brooksia lacromae* | Salp sequence | Retrieved from GenBank |
| KR057222.1 | *Brooksia lacromae* | Salp sequence | Retrieved from GenBank |
| MZ333593.1 | *Salpa younti* | Salp sequence | Retrieved from GenBank |
| AB859889.1 | *Thalia longicauda* | Salp sequence | Retrieved from GenBank |
| AB013011.1 | *Pyrosoma atlanticum* | Outgroup sequence | Retrieved from GenBank |
| AB013012.1 | *Doliolum nationalis* | Outgroup sequence | Retrieved from GenBank |
| AB013017.1 | *Ciona intestinalis* | Outgroup sequence | Retrieved from GenBank |
| D14366.1 | *Thalia democratica* | Outgroup sequence | Retrieved from GenBank |
| FM244861.1 | *Doliolum denticulatum* | Outgroup sequence | Retrieved from GenBank |
| FM244862.1 | *Pyrosoma godeauxi* | Outgroup sequence | Retrieved from GenBank |
| FM244863.1 | *Pyrosomella verticillata* | Outgroup sequence | Retrieved from GenBank |
| L12426.2 | *Molgula manhattensis* | Outgroup sequence | Retrieved from GenBank |
| M91181.1 | *Echinorhinus cookei* | Outgroup sequence | Retrieved from GenBank |
| M97574.1 | *Myxine glutinosa* | Outgroup sequence | Retrieved from GenBank |
| M97571.1 | *Branchiostoma floridae* | Outgroup sequence | Retrieved from GenBank |
| AB013014.1 | *Oikopleura dioica* | Outgroup sequence | Retrieved from GenBank |
| AY903925.1 | *Halocynthia igaboja* | Outgroup sequence | Retrieved from GenBank |
| FM244840.1 | *Clavelina meridionalis* | Outgroup sequence | Retrieved from GenBank |
| FM244841.1 | *Pycnoclavella aff. detorta* | Outgroup sequence | Retrieved from GenBank |
| AB075543.1 | *Megalodicopia hians* | Outgroup sequence | Retrieved from GenBank |
| L12378.2 | *Ascidia ceratodes* | Outgroup sequence | Retrieved from GenBank |
| AJ250778.1 | *Ciona intestinalis* | Outgroup sequence | Retrieved from GenBank |
| AB104873.1 | *Perophora sagamiensis* | Outgroup sequence | Retrieved from GenBank |
| AF165821.2 | *Chelyosoma siboja* | Outgroup sequence | Retrieved from GenBank |
| HQ015385.1 | *Pyrosoma atlanticum* | Outgroup sequence | Retrieved from GenBank |
| HQ015384.1 | *Pyrosoma godeauxi* | Outgroup sequence | Retrieved from GenBank |
| HQ015383.1 | *Pyrosomella verticillata* | Outgroup sequence | Retrieved from GenBank |
| HQ015382.1 | *Pyrosomella verticillata* | Outgroup sequence | Retrieved from GenBank |
| HQ015381.1 | *Pyrosoma atlanticum* | Outgroup sequence | Retrieved from GenBank |
| HQ015380.1 | *Pyrosomella verticillata* | Outgroup sequence | Retrieved from GenBank |
| HQ015379.1 | *Pyrostremma spinosum* | Outgroup sequence | Retrieved from GenBank |
| HQ015376.1 | *Doliolum denticulatum* | Outgroup sequence | Retrieved from GenBank |

SM Table 2. Dorsoventral zooid-stolon angle measurements.

| **Filename** | **Source** | **Species** | **Specimen_type** | **Specimen** | **Architecture** | **Trait** | **State** | **Notes** |
| --- | --- | --- | --- | --- | --- | --- | --- | --- |
| **DSC_0482.jpg** | SSW22-NH5-MOC4-Net3-Pegea socia | *Pegea socia* | FreshSpecimen | 13 | Transversal | DV_Zooid.stolon.angle | 88.60 |  |
| **IMG0007** | Kona April 2022 | *Cyclosalpa affinis* | FreshSpecimen | D15-Caff-B-1 | Whorl | DV_Zooid.stolon.angle | 90.28 |  |
| **IMG0007** | Kona April 2022 | *Cyclosalpa affinis* | FreshSpecimen | D15-Caff-B-1 | Whorl | DV_Zooid.stolon.angle | 86.68 |  |
| **IMG0007** | Kona April 2022 | *Cyclosalpa affinis* | FreshSpecimen | D15-Caff-B-1 | Whorl | DV_Zooid.stolon.angle | 92.28 |  |
| **IMG0016** | Kona April 2022 | *Cyclosalpa polae* | FreshSpecimen | D15-Cpol-B-1 | Cluster | DV_Zooid.stolon.angle | 82.75 |  |
| **IMG0016** | Kona April 2022 | *Cyclosalpa polae* | FreshSpecimen | D15-Cpol-B-1 | Cluster | DV_Zooid.stolon.angle | 85.27 |  |
| **IMG0016** | Kona April 2022 | *Cyclosalpa polae* | FreshSpecimen | D15-Cpol-B-1 | Cluster | DV_Zooid.stolon.angle | 60.00 |  |
| **IMG0062** | Kona April 2022 | *Cyclosalpa sewelli* | FreshSpecimen | D16-Csew-B-1 | Cluster | DV_Zooid.stolon.angle | 78.95 |  |
| **IMG0062** | Kona April 2022 | *Cyclosalpa sewelli* | FreshSpecimen | D16-Csew-B-1 | Cluster | DV_Zooid.stolon.angle | 75.79 |  |
| **IMG0062** | Kona April 2022 | *Cyclosalpa sewelli* | FreshSpecimen | D16-Csew-B-1 | Cluster | DV_Zooid.stolon.angle | 52.58 |  |
| **IMG0089** | Kona April 2022 | *Cyclosalpa affinis* | FreshSpecimen | D17-Caff-B-1 | Whorl | DV_Zooid.stolon.angle | 85.15 |  |
| **IMG0089** | Kona April 2022 | *Cyclosalpa affinis* | FreshSpecimen | D17-Caff-B-1 | Whorl | DV_Zooid.stolon.angle | 86.91 |  |
| **IMG0089** | Kona April 2022 | *Cyclosalpa affinis* | FreshSpecimen | D17-Caff-B-1 | Whorl | DV_Zooid.stolon.angle | 88.00 |  |
| **IMG0097** | Kona April 2022 | *Cyclosalpa affinis* | FreshSpecimen | D17-Caff-B-2 | Whorl | DV_Zooid.stolon.angle | 86.09 |  |
| **IMG0097** | Kona April 2022 | *Cyclosalpa affinis* | FreshSpecimen | D17-Caff-B-2 | Whorl | DV_Zooid.stolon.angle | 89.75 |  |
| **IMG0097** | Kona April 2022 | *Cyclosalpa affinis* | FreshSpecimen | D17-Caff-B-2 | Whorl | DV_Zooid.stolon.angle | 86.62 |  |
| **Img0108** | Kona April 2022 | *Iasis cylindrica* | FreshSpecimen | D18-Icyl-B-1 | Linear | DV_Zooid.stolon.angle | 19.53 |  |
| **Img0108** | Kona April 2022 | *Iasis cylindrica* | FreshSpecimen | D18-Icyl-B-1 | Linear | DV_Zooid.stolon.angle | 16.98 |  |
| **Img0108** | Kona April 2022 | *Iasis cylindrica* | FreshSpecimen | D18-Icyl-B-1 | Linear | DV_Zooid.stolon.angle | 20.13 |  |
| **Img0124** | Kona April 2022 | *Thalia longicauda* | FreshSpecimen | D20-Tlon-B-1 | Oblique | DV_Zooid.stolon.angle | 46.15 |  |
| **Img0124** | Kona April 2022 | *Thalia longicauda* | FreshSpecimen | D20-Tlon-B-1 | Oblique | DV_Zooid.stolon.angle | 50.12 |  |
| **Img0124** | Kona April 2022 | *Thalia longicauda* | FreshSpecimen | D20-Tlon-B-1 | Oblique | DV_Zooid.stolon.angle | 49.30 |  |
| **DSC_0904** | Kona April 2022 | *Cyclosalpa bakeri* | FreshSpecimen | D24-Cbak-B-1 | Whorl | DV_Zooid.stolon.angle | 87.54 |  |
| **DSC_0904** | Kona April 2022 | *Cyclosalpa bakeri* | FreshSpecimen | D24-Cbak-B-1 | Whorl | DV_Zooid.stolon.angle | 89.98 |  |
| **DSC_0904** | Kona April 2022 | *Cyclosalpa bakeri* | FreshSpecimen | D24-Cbak-B-1 | Whorl | DV_Zooid.stolon.angle | 88.47 |  |
| **DSC_0924** | Kona April 2022 | *Soestia zonaria* | FreshSpecimen | D24-Szon-B-1 | Linear | DV_Zooid.stolon.angle | 1.56 |  |
| **DSC_0924** | Kona April 2022 | *Soestia zonaria* | FreshSpecimen | D24-Szon-B-1 | Linear | DV_Zooid.stolon.angle | 1.89 |  |
| **DSC_0924** | Kona April 2022 | *Soestia zonaria* | FreshSpecimen | D24-Szon-B-1 | Linear | DV_Zooid.stolon.angle | 1.84 |  |
| **DSC_0951** | Kona April 2022 | *Salpa fusiformis* | FreshSpecimen | D24-Sfus-B-1 | Linear | DV_Zooid.stolon.angle | 17.80 |  |
| **DSC_0951** | Kona April 2022 | *Salpa fusiformis* | FreshSpecimen | D24-Sfus-B-1 | Linear | DV_Zooid.stolon.angle | 18.77 |  |
| **DSC_0977** | Kona April 2022 | *Cyclosalpa bakeri* | FreshSpecimen | D26-Cbak-B-1 | Whorl | DV_Zooid.stolon.angle | 88.85 |  |
| **DSC_0977** | Kona April 2022 | *Cyclosalpa bakeri* | FreshSpecimen | D26-Cbak-B-1 | Whorl | DV_Zooid.stolon.angle | 85.27 |  |
| **DSC_0977** | Kona April 2022 | *Cyclosalpa bakeri* | FreshSpecimen | D26-Cbak-B-1 | Whorl | DV_Zooid.stolon.angle | 86.44 |  |
| **DSC_0970** | Kona April 2022 | *Pegea sp.* | FreshSpecimen | D25-Psp-B-1 | Transversal | DV_Zooid.stolon.angle | 87.46 |  |
| **DSC_0970** | Kona April 2022 | *Pegea sp.* | FreshSpecimen | D25-Psp-B-1 | Transversal | DV_Zooid.stolon.angle | 88.44 |  |
| **DSC_0970** | Kona April 2022 | *Pegea sp.* | FreshSpecimen | D25-Psp-B-1 | Transversal | DV_Zooid.stolon.angle | 89.02 |  |
| **DSC_0964** | Kona April 2022 | *Cyclosalpa quadriluminis* | FreshSpecimen | D25-Cqua-B-1 | Whorl | DV_Zooid.stolon.angle | 87.38 |  |
| **DSC_0964** | Kona April 2022 | *Cyclosalpa quadriluminis* | FreshSpecimen | D25-Cqua-B-1 | Whorl | DV_Zooid.stolon.angle | 83.80 |  |
| **DSC_0964** | Kona April 2022 | *Cyclosalpa quadriluminis* | FreshSpecimen | D25-Cqua-B-1 | Whorl | DV_Zooid.stolon.angle | 85.64 |  |
| **Img0048** | Kona April 2022 | *Salpa aspera* | FreshSpecimen | D20-Sasp-B-1 | Linear | DV_Zooid.stolon.angle | 19.27 |  |
| **Img0048** | Kona April 2022 | *Salpa aspera* | FreshSpecimen | D20-Sasp-B-1 | Linear | DV_Zooid.stolon.angle | 16.91 |  |
| **Img0048** | Kona April 2022 | *Salpa aspera* | FreshSpecimen | D20-Sasp-B-1 | Linear | DV_Zooid.stolon.angle | 7.50 |  |
| **Img0132** | Kona April 2022 | *Iasis cylindrica* | FreshSpecimen | D22-Icyl-B-2 | Linear | DV_Zooid.stolon.angle | 17.14 |  |
| **Img0132** | Kona April 2022 | *Iasis cylindrica* | FreshSpecimen | D22-Icyl-B-2 | Linear | DV_Zooid.stolon.angle | 20.47 |  |
| **Img0132** | Kona April 2022 | *Iasis cylindrica* | FreshSpecimen | D22-Icyl-B-2 | Linear | DV_Zooid.stolon.angle | 12.76 |  |
| **Img0152** | Kona April 2022 | *Ritteriella amboinensis* | FreshSpecimen | D22-Ramb-B-1 | Bipinnate | DV_Zooid.stolon.angle | 9.84 | lateral view of zooid, used endostyle and tip of viscerae for angle |
| **Img0152** | Kona April 2022 | *Ritteriella amboinensis* | FreshSpecimen | D22-Ramb-B-1 | Bipinnate | DV_Zooid.stolon.angle | 8.56 | lateral view of zooid, used endostyle and tip of viscerae for angle |
| **Img0152** | Kona April 2022 | *Ritteriella amboinensis* | FreshSpecimen | D22-Ramb-B-1 | Bipinnate | DV_Zooid.stolon.angle | 10.97 | lateral view of zooid, used endostyle and tip of viscerae for angle |
| **KonaJJ22-020** | Kona June-July 2022 | *Helicosalpa younti* | FreshSpecimen | D27-Hyou-B-1 | Helical | DV_Zooid.stolon.angle | 82.20 |  |
| **KonaJJ22-020** | Kona June-July 2022 | *Helicosalpa younti* | FreshSpecimen | D27-Hyou-B-1 | Helical | DV_Zooid.stolon.angle | 79.67 |  |
| **KonaJJ22-020** | Kona June-July 2022 | *Helicosalpa younti* | FreshSpecimen | D27-Hyou-B-1 | Helical | DV_Zooid.stolon.angle | 89.92 |  |
| **KonaJJ22-053** | Kona June-July 2022 | *Ritteriella amboinensis* | FreshSpecimen | D27-Ramb-B-1 | Bipinnate | DV_Zooid.stolon.angle | 8.34 | lateral view of zooid, used endostyle and tip of viscerae for angle |
| **KonaJJ22-053** | Kona June-July 2022 | *Ritteriella amboinensis* | FreshSpecimen | D27-Ramb-B-1 | Bipinnate | DV_Zooid.stolon.angle | 9.38 | lateral view of zooid, used endostyle and tip of viscerae for angle |
| **KonaJJ22-053** | Kona June-July 2022 | *Ritteriella amboinensis* | FreshSpecimen | D27-Ramb-B-1 | Bipinnate | DV_Zooid.stolon.angle | 8.30 | lateral view of zooid, used endostyle and tip of viscerae for angle |
| **KonaJJ22-081** | Kona June-July 2022 | *Pegea sp.* | FreshSpecimen | D27-Psp-B-1 | Transversal | DV_Zooid.stolon.angle | 90.06 |  |
| **KonaJJ22-081** | Kona June-July 2022 | *Pegea sp.* | FreshSpecimen | D27-Psp-B-1 | Transversal | DV_Zooid.stolon.angle | 88.86 |  |
| **KonaJJ22-081** | Kona June-July 2022 | *Pegea sp.* | FreshSpecimen | D27-Psp-B-1 | Transversal | DV_Zooid.stolon.angle | 86.56 |  |
| **KonaJJ22-109** | Kona June-July 2022 | *Cyclosalpa sewelli* | FreshSpecimen | D28-Csew-B-1 | Cluster | DV_Zooid.stolon.angle | 85.20 |  |
| **KonaJJ22-109** | Kona June-July 2022 | *Cyclosalpa sewelli* | FreshSpecimen | D28-Csew-B-1 | Cluster | DV_Zooid.stolon.angle | 80.91 | lateral view of zooid, used endostyle and tip of viscerae for angle |
| **KonaJJ22-109** | Kona June-July 2022 | *Cyclosalpa sewelli* | FreshSpecimen | D28-Csew-B-1 | Cluster | DV_Zooid.stolon.angle | 84.90 | lateral view of zooid, used endostyle and tip of viscerae for angle |
| **KonaJJ22-116** | Kona June-July 2022 | *Brooksia rostrata* | FreshSpecimen | D28-Bros-B-1 | Bipinnate | DV_Zooid.stolon.angle | 61.60 | lateral view of zooid, used line between base and tip of the endostyle |
| **KonaJJ22-116** | Kona June-July 2022 | *Brooksia rostrata* | FreshSpecimen | D28-Bros-B-1 | Bipinnate | DV_Zooid.stolon.angle | 59.01 | lateral view of zooid, used line between base and tip of the endostyle |
| **KonaJJ22-116** | Kona June-July 2022 | *Brooksia rostrata* | FreshSpecimen | D28-Bros-B-1 | Bipinnate | DV_Zooid.stolon.angle | 50.16 | lateral view of zooid, used line between base and tip of the endostyle |
| **KonaJJ22-146** | Kona June-July 2022 | *Salpa maxima* | FreshSpecimen | D29-Smax-B-1 | Linear | DV_Zooid.stolon.angle | 15.06 |  |
| **KonaJJ22-146** | Kona June-July 2022 | *Salpa maxima* | FreshSpecimen | D29-Smax-B-1 | Linear | DV_Zooid.stolon.angle | 18.00 |  |
| **KonaJJ22-218** | Kona June-July 2022 | *Cyclosalpa bakeri* | FreshSpecimen | D30-Cbak-B-1 | Whorl | DV_Zooid.stolon.angle | 91.15 |  |
| **KonaJJ22-218** | Kona June-July 2022 | *Cyclosalpa bakeri* | FreshSpecimen | D30-Cbak-B-1 | Whorl | DV_Zooid.stolon.angle | 88.19 |  |
| **KonaJJ22-218** | Kona June-July 2022 | *Cyclosalpa bakeri* | FreshSpecimen | D30-Cbak-B-1 | Whorl | DV_Zooid.stolon.angle | 80.19 | lateral view of zooid, used endostyle and tip of viscerae for angle |
| **KonaJJ22-372** | Kona June-July 2022 | *Ritteriella retracta* | FreshSpecimen | D31-Rsp-B-1 | Bipinnate | DV_Zooid.stolon.angle | 14.43 | lateral view of zooid, used line between base and tip of the endostyle |
| **KonaJJ22-372** | Kona June-July 2022 | *Ritteriella retracta* | FreshSpecimen | D31-Rsp-B-1 | Bipinnate | DV_Zooid.stolon.angle | 10.88 | lateral view of zooid, used line between base and tip of the endostyle |
| **KonaJJ22-372** | Kona June-July 2022 | *Ritteriella retracta* | FreshSpecimen | D31-Rsp-B-1 | Bipinnate | DV_Zooid.stolon.angle | 10.36 | lateral view of zooid, used line between base and tip of the endostyle |
| **KonaJJ22-396** | Kona June-July 2022 | *Cyclosalpa quadriluminis* | FreshSpecimen | D31-Cqua-B-1 | Whorl | DV_Zooid.stolon.angle | 88.36 |  |
| **KonaJJ22-396** | Kona June-July 2022 | *Cyclosalpa quadriluminis* | FreshSpecimen | D31-Cqua-B-1 | Whorl | DV_Zooid.stolon.angle | 89.66 |  |
| **KonaJJ22-396** | Kona June-July 2022 | *Cyclosalpa quadriluminis* | FreshSpecimen | D31-Cqua-B-1 | Whorl | DV_Zooid.stolon.angle | 83.53 |  |
| **KonaJJ22-412** | Kona June-July 2022 | *Salpa maxima* | FreshSpecimen | D31-Smax-B-1 | Linear | DV_Zooid.stolon.angle | 21.60 |  |
| **KonaJJ22-412** | Kona June-July 2022 | *Salpa maxima* | FreshSpecimen | D31-Smax-B-1 | Linear | DV_Zooid.stolon.angle | 19.09 |  |
| **KonaJJ22-408** | Kona June-July 2022 | *Salpa maxima* | FreshSpecimen | D31-Smax-B-1 | Linear | DV_Zooid.stolon.angle | 28.88 |  |
| **KonaJJ22-481** | Kona June-July 2022 | *Pegea sp.* | FreshSpecimen | D31-Psp-B-1 | Transversal | DV_Zooid.stolon.angle | 87.10 |  |
| **KonaJJ22-481** | Kona June-July 2022 | *Pegea sp.* | FreshSpecimen | D31-Psp-B-1 | Transversal | DV_Zooid.stolon.angle | 89.76 |  |
| **KonaJJ22-481** | Kona June-July 2022 | *Pegea sp.* | FreshSpecimen | D31-Psp-B-1 | Transversal | DV_Zooid.stolon.angle | 87.68 |  |
| **KonaJJ22-494** | Kona June-July 2022 | *Ihlea punctata* | FreshSpecimen | D32-Ipun-B-1 | Linear | DV_Zooid.stolon.angle | 23.11 |  |
| **KonaJJ22-494** | Kona June-July 2022 | *Ihlea punctata* | FreshSpecimen | D32-Ipun-B-1 | Linear | DV_Zooid.stolon.angle | 25.13 |  |
| **KonaJJ22-494** | Kona June-July 2022 | *Ihlea punctata* | FreshSpecimen | D32-Ipun-B-1 | Linear | DV_Zooid.stolon.angle | 28.85 |  |
| **KonaJJ22-525** | Kona June-July 2022 | *Iasis cylindrica* | FreshSpecimen | D32-Icyl-B-3 | Linear | DV_Zooid.stolon.angle | 24.01 | young chain |
| **KonaJJ22-525** | Kona June-July 2022 | *Iasis cylindrica* | FreshSpecimen | D32-Icyl-B-3 | Linear | DV_Zooid.stolon.angle | 26.97 | young chain |
| **KonaJJ22-525** | Kona June-July 2022 | *Iasis cylindrica* | FreshSpecimen | D32-Icyl-B-3 | Linear | DV_Zooid.stolon.angle | 29.15 | young chain |
| **KonaJJ22-551** | Kona June-July 2022 | *Ritteriella retracta* | FreshSpecimen | D32-Rsp-B-1 | Bipinnate | DV_Zooid.stolon.angle | 13.22 | lateral view of zooid, used line between base and tip of the endostyle |
| **KonaJJ22-551** | Kona June-July 2022 | *Ritteriella retracta* | FreshSpecimen | D32-Rsp-B-1 | Bipinnate | DV_Zooid.stolon.angle | 11.16 | lateral view of zooid, used line between base and tip of the endostyle |
| **KonaJJ22-551** | Kona June-July 2022 | *Ritteriella retracta* | FreshSpecimen | D32-Rsp-B-1 | Bipinnate | DV_Zooid.stolon.angle | 7.66 | lateral view of zooid, used line between base and tip of the endostyle |
| **KonaJJ22-625** | Kona September 2022 | *Brooksia rostrata* | FreshSpecimen | D33-Bros-B-1 | Bipinnate | DV_Zooid.stolon.angle | 51.78 | lateral view of zooid, used line between base and tip of the endostyle |
| **KonaJJ22-625** | Kona September 2022 | *Brooksia rostrata* | FreshSpecimen | D33-Bros-B-1 | Bipinnate | DV_Zooid.stolon.angle | 55.89 | lateral view of zooid, used line between base and tip of the endostyle |
| **KonaJJ22-625** | Kona September 2022 | *Brooksia rostrata* | FreshSpecimen | D33-Bros-B-1 | Bipinnate | DV_Zooid.stolon.angle | 52.18 | lateral view of zooid, used line between base and tip of the endostyle |
| **Kona09-22-700** | Kona September 2022 | *Cyclosalpa quadriluminis* | FreshSpecimen | D36-Cqua-B-1 | Whorl | DV_Zooid.stolon.angle | 84.70 | used viscera for zooid axis |
| **Kona09-22-700** | Kona September 2022 | *Cyclosalpa quadriluminis* | FreshSpecimen | D36-Cqua-B-1 | Whorl | DV_Zooid.stolon.angle | 89.97 | used viscera for zooid axis |
| **Kona09-22-700** | Kona September 2022 | *Cyclosalpa quadriluminis* | FreshSpecimen | D36-Cqua-B-1 | Whorl | DV_Zooid.stolon.angle | 87.58 | used viscera for zooid axis |
| **Kona09-22-717** | Kona September 2022 | *Soestia zonaria* | FreshSpecimen | D37-Szon-B-1 | Linear | DV_Zooid.stolon.angle | 3.14 |  |
| **Kona09-22-717** | Kona September 2022 | *Soestia zonaria* | FreshSpecimen | D37-Szon-B-1 | Linear | DV_Zooid.stolon.angle | 0.29 |  |
| **Kona09-22-717** | Kona September 2022 | *Soestia zonaria* | FreshSpecimen | D37-Szon-B-1 | Linear | DV_Zooid.stolon.angle | 0.18 |  |
| **Kona09-22-787** | Kona September 2022 | *Metcalfina hexagona* | FreshSpecimen | D39-Mhex-B-1 | Linear | DV_Zooid.stolon.angle | 16.15 |  |
| **Kona09-22-787** | Kona September 2022 | *Metcalfina hexagona* | FreshSpecimen | D39-Mhex-B-1 | Linear | DV_Zooid.stolon.angle | 15.60 |  |
| **Kona09-22-787** | Kona September 2022 | *Metcalfina hexagona* | FreshSpecimen | D39-Mhex-B-1 | Linear | DV_Zooid.stolon.angle | 13.15 |  |
| **Kona09-22-808** | Kona September 2022 | *Cyclosalpa polae* | FreshSpecimen | D40-Cpol-B-1 | Cluster | DV_Zooid.stolon.angle | 52.76 | used viscera for zooid axis |
| **Kona09-22-808** | Kona September 2022 | *Cyclosalpa polae* | FreshSpecimen | D40-Cpol-B-1 | Cluster | DV_Zooid.stolon.angle | 71.27 | used viscera for zooid axis |
| **Kona09-22-808** | Kona September 2022 | *Cyclosalpa polae* | FreshSpecimen | D40-Cpol-B-1 | Cluster | DV_Zooid.stolon.angle | 68.78 | used viscera for zooid axis |
| **Kona09-22-848** | Kona September 2022 | *Brooksia rostrata* | FreshSpecimen | D41-Bros-B-1 | Bipinnate | DV_Zooid.stolon.angle | 19.71 | lateral view of zooid, used line between base and tip of the endostyle |
| **Kona09-22-848** | Kona September 2022 | *Brooksia rostrata* | FreshSpecimen | D41-Bros-B-1 | Bipinnate | DV_Zooid.stolon.angle | 23.48 | lateral view of zooid, used line between base and tip of the endostyle |
| **Kona09-22-848** | Kona September 2022 | *Brooksia rostrata* | FreshSpecimen | D41-Bros-B-1 | Bipinnate | DV_Zooid.stolon.angle | 20.91 | lateral view of zooid, used line between base and tip of the endostyle |
| **Kona09-22-838** | Kona September 2022 | *Iasis cylindrica* | FreshSpecimen | D40-Icyl-B-1 | Linear | DV_Zooid.stolon.angle | 26.09 |  |
| **Kona09-22-838** | Kona September 2022 | *Iasis cylindrica* | FreshSpecimen | D40-Icyl-B-1 | Linear | DV_Zooid.stolon.angle | 29.63 |  |
| **Kona09-22-838** | Kona September 2022 | *Iasis cylindrica* | FreshSpecimen | D40-Icyl-B-1 | Linear | DV_Zooid.stolon.angle | 24.23 |  |
| **Kona09-22-826** | Kona September 2022 | *Salpa maxima* | FreshSpecimen | D40-Smax-B-1 | Linear | DV_Zooid.stolon.angle | 22.50 |  |
| **Kona09-22-826** | Kona September 2022 | *Salpa maxima* | FreshSpecimen | D40-Smax-B-1 | Linear | DV_Zooid.stolon.angle | 33.05 |  |
| **Kona09-22-826** | Kona September 2022 | *Salpa maxima* | FreshSpecimen | D40-Smax-B-1 | Linear | DV_Zooid.stolon.angle | 24.91 |  |
| **Kona09-22-914** | Kona September 2022 | *Helicosalpa virgula* | FreshSpecimen | D43-Hvir-B-1 | Helical | DV_Zooid.stolon.angle | 89.44 |  |
| **Kona09-22-914** | Kona September 2022 | *Helicosalpa virgula* | FreshSpecimen | D43-Hvir-B-1 | Helical | DV_Zooid.stolon.angle | 89.79 |  |
| **Kona09-22-914** | Kona September 2022 | *Helicosalpa virgula* | FreshSpecimen | D43-Hvir-B-1 | Helical | DV_Zooid.stolon.angle | 87.55 |  |
| **Kona09-22-887** | Kona September 2022 | *Thalia longicauda* | FreshSpecimen | D42-Tlon-B-1 | Oblique | DV_Zooid.stolon.angle | 55.87 |  |
| **Kona09-22-887** | Kona September 2022 | *Thalia longicauda* | FreshSpecimen | D42-Tlon-B-1 | Oblique | DV_Zooid.stolon.angle | 56.27 |  |
| **Kona09-22-887** | Kona September 2022 | *Thalia longicauda* | FreshSpecimen | D42-Tlon-B-1 | Oblique | DV_Zooid.stolon.angle | 47.59 |  |
| **KonaMay23_0049** | Kona May 2023 | *Pegea sp.* | FreshSpecimen | D46-Psp-B-1 | Transversal | DV_Zooid.stolon.angle | 88.07 | young zooids |
| **KonaMay23_0049** | Kona May 2023 | *Pegea sp.* | FreshSpecimen | D46-Psp-B-1 | Transversal | DV_Zooid.stolon.angle | 88.29 | young zooids |
| **KonaMay23_0049** | Kona May 2023 | *Pegea sp.* | FreshSpecimen | D46-Psp-B-1 | Transversal | DV_Zooid.stolon.angle | 85.85 | young zooids |
| **KonaMay23_0040** | Kona May 2023 | *Soestia zonaria* | FreshSpecimen | D47-Szon-B-1 | Linear | DV_Zooid.stolon.angle | 3.55 | dead post anesthetic, insides shrivelled |
| **KonaMay23_0040** | Kona May 2023 | *Soestia zonaria* | FreshSpecimen | D47-Szon-B-1 | Linear | DV_Zooid.stolon.angle | 1.06 | dead post anesthetic, insides shrivelled |
| **KonaMay23_0040** | Kona May 2023 | *Soestia zonaria* | FreshSpecimen | D47-Szon-B-1 | Linear | DV_Zooid.stolon.angle | 1.46 | dead post anesthetic, insides shrivelled |
| **KonaMay23_0090** | Kona May 2023 | *Cyclosalpa polae* | FreshSpecimen | D48-Cpol-B-1 | Cluster | DV_Zooid.stolon.angle | 89.30 |  |
| **KonaMay23_0090** | Kona May 2023 | *Cyclosalpa polae* | FreshSpecimen | D48-Cpol-B-1 | Cluster | DV_Zooid.stolon.angle | 89.19 |  |
| **KonaMay23_0090** | Kona May 2023 | *Cyclosalpa polae* | FreshSpecimen | D48-Cpol-B-1 | Cluster | DV_Zooid.stolon.angle | 88.64 | lateral view of zooid, used endostyle and tip of viscerae for angle |
| **KonaMay23_0068** | Kona May 2023 | *Thalia cicar* | FreshSpecimen | D48-Tcic-B-2 | Oblique | DV_Zooid.stolon.angle | 47.66 |  |
| **KonaMay23_0068** | Kona May 2023 | *Thalia cicar* | FreshSpecimen | D48-Tcic-B-2 | Oblique | DV_Zooid.stolon.angle | 48.05 |  |
| **KonaMay23_0068** | Kona May 2023 | *Thalia cicar* | FreshSpecimen | D48-Tcic-B-2 | Oblique | DV_Zooid.stolon.angle | 49.04 |  |
| **KonaMay23_0105** | Kona May 2023 | *Cyclosalpa polae* | FreshSpecimen | D51-Cpol-B-3 | Cluster | DV_Zooid.stolon.angle | 88.27 |  |
| **KonaMay23_0105** | Kona May 2023 | *Cyclosalpa polae* | FreshSpecimen | D51-Cpol-B-3 | Cluster | DV_Zooid.stolon.angle | 87.03 | lateral view of zooid, used endostyle and tip of viscerae for angle |
| **KonaMay23_0105** | Kona May 2023 | *Cyclosalpa polae* | FreshSpecimen | D51-Cpol-B-3 | Cluster | DV_Zooid.stolon.angle | 89.19 | lateral view of zooid, used endostyle and tip of viscerae for angle |
| **D27-Cpin-B-1.mp4 07:32:44:20** | Kona June-July 2022 | *Cyclosalpa pinnata* | FreshSpecimen | D27-Cpin-B-1 (GoPro) | Cluster | DV_Zooid.stolon.angle | 89.59 | Used viscera for zooid axis |
| **D27-Cpin-B-1.mp4 07:32:44:20** | Kona June-July 2022 | *Cyclosalpa pinnata* | FreshSpecimen | D27-Cpin-B-1 (GoPro) | Cluster | DV_Zooid.stolon.angle | 86.13 | Used viscera for zooid axis |
| **D27-Cpin-B-1.mp4 07:32:44:20** | Kona June-July 2022 | *Cyclosalpa pinnata* | FreshSpecimen | D27-Cpin-B-1 (GoPro) | Cluster | DV_Zooid.stolon.angle | 89.25 | Used viscera for zooid axis |
| **tape3_scale41_14 00:02** | LJL Vegas Clips | *Salpa thompsoni* | FreshSpecimen | Vegas tape3_scale41_14 | Linear | DV_Zooid.stolon.angle | 18.44 |  |
| **tape3_scale41_14 00:02** | LJL Vegas Clips | *Salpa thompsoni* | FreshSpecimen | Vegas tape3_scale41_14 | Linear | DV_Zooid.stolon.angle | 17.09 |  |
| **tape3_scale41_14 00:02** | LJL Vegas Clips | *Salpa thompsoni* | FreshSpecimen | Vegas tape3_scale41_14 | Linear | DV_Zooid.stolon.angle | 17.65 |  |
| **Thetys vagina - Salp Chain / Carmel River - 9/14/14 00:24** | <https://vimeo.com/106143426> | *Thetys vagina* | UWvideo | 16 | Oblique | DV_Zooid.stolon.angle | 43.27 |  |
| **Thetys vagina - Salp Chain / Carmel River - 9/14/14 00:24** | <https://vimeo.com/106143426> | *Thetys vagina* | UWvideo | 16 | Oblique | DV_Zooid.stolon.angle | 40.96 |  |
| **Thetys vagina - Salp Chain / Carmel River - 9/14/14 00:24** | <https://vimeo.com/106143426> | *Thetys vagina* | UWvideo | 16 | Oblique | DV_Zooid.stolon.angle | 37.23 |  |
| **Shale_Thalia_democratica_1** | <https://www.naturepl.com/stock-photo--thalia-democratica-salp-chain-deep-sea-atlantic-image01126842.html> | *Thalia democratica* | UWphoto | 3 | Oblique | DV_Zooid.stolon.angle | 64.25 |  |
| **Shale_Thalia_democratica_1** | <https://www.naturepl.com/stock-photo--thalia-democratica-salp-chain-deep-sea-atlantic-image01126842.html> | *Thalia democratica* | UWphoto | 3 | Oblique | DV_Zooid.stolon.angle | 63.93 |  |
| **Shale_Thalia_democratica_1** | <https://www.naturepl.com/stock-photo--thalia-democratica-salp-chain-deep-sea-atlantic-image01126842.html> | *Thalia democratica* | UWphoto | 3 | Oblique | DV_Zooid.stolon.angle | 56.16 |  |
| [**https://scontent.fhio3-1.fna.fbcdn.net/v/t1.6435-9/97992579_2458196494426958_3526566602721984512_n.jpg?_nc_cat=110&ccb=1-7&_nc_sid=730e14&_nc_ohc=axtnPRePZpoAX9flyaX&_nc_ht=scontent.fhio3-1.fna&oh=00_AfDleilkU4rH0gAz1rw9hmNopKKBt-K3ny7iOWimVWtqKw&oe=64987DC8**](https://scontent.fhio3-1.fna.fbcdn.net/v/t1.6435-9/97992579_2458196494426958_3526566602721984512_n.jpg?_nc_cat=110&ccb=1-7&_nc_sid=730e14&_nc_ohc=axtnPRePZpoAX9flyaX&_nc_ht=scontent.fhio3-1.fna&oh=00_AfDleilkU4rH0gAz1rw9hmNopKKBt-K3ny7iOWimVWtqKw&oe=64987DC8) | <https://www.facebook.com/blackwaterdive/posts/seacam1970-aggregate-zooid-of-pegea-bicaudata-its-common-species-off-shore-of-ki/2457547687825172/> | *Pegea bicaudata* | UWphoto | 17 | Transversal | DV_Zooid.stolon.angle | 88.64 |  |
| [**https://scontent.fhio3-1.fna.fbcdn.net/v/t1.6435-9/97992579_2458196494426958_3526566602721984512_n.jpg?_nc_cat=110&ccb=1-7&_nc_sid=730e14&_nc_ohc=axtnPRePZpoAX9flyaX&_nc_ht=scontent.fhio3-1.fna&oh=00_AfDleilkU4rH0gAz1rw9hmNopKKBt-K3ny7iOWimVWtqKw&oe=64987DC8**](https://scontent.fhio3-1.fna.fbcdn.net/v/t1.6435-9/97992579_2458196494426958_3526566602721984512_n.jpg?_nc_cat=110&ccb=1-7&_nc_sid=730e14&_nc_ohc=axtnPRePZpoAX9flyaX&_nc_ht=scontent.fhio3-1.fna&oh=00_AfDleilkU4rH0gAz1rw9hmNopKKBt-K3ny7iOWimVWtqKw&oe=64987DC8) | <https://www.facebook.com/blackwaterdive/posts/seacam1970-aggregate-zooid-of-pegea-bicaudata-its-common-species-off-shore-of-ki/2457547687825172/> | *Pegea bicaudata* | UWphoto | 17 | Transversal | DV_Zooid.stolon.angle | 87.50 |  |
| [**https://scontent.fhio3-1.fna.fbcdn.net/v/t1.6435-9/97992579_2458196494426958_3526566602721984512_n.jpg?_nc_cat=110&ccb=1-7&_nc_sid=730e14&_nc_ohc=axtnPRePZpoAX9flyaX&_nc_ht=scontent.fhio3-1.fna&oh=00_AfDleilkU4rH0gAz1rw9hmNopKKBt-K3ny7iOWimVWtqKw&oe=64987DC8**](https://scontent.fhio3-1.fna.fbcdn.net/v/t1.6435-9/97992579_2458196494426958_3526566602721984512_n.jpg?_nc_cat=110&ccb=1-7&_nc_sid=730e14&_nc_ohc=axtnPRePZpoAX9flyaX&_nc_ht=scontent.fhio3-1.fna&oh=00_AfDleilkU4rH0gAz1rw9hmNopKKBt-K3ny7iOWimVWtqKw&oe=64987DC8) | <https://www.facebook.com/blackwaterdive/posts/seacam1970-aggregate-zooid-of-pegea-bicaudata-its-common-species-off-shore-of-ki/2457547687825172/> | *Pegea bicaudata* | UWphoto | 17 | Transversal | DV_Zooid.stolon.angle | 89.43 |  |
| **DSC_0482.jpg** | SSW22-NH5-MOC4-Net3-Pegea socia | *Pegea socia* | FreshSpecimen | SSW22-NH5-MOC4-Net3 | Transversal | DV_Zooid.stolon.angle | 87.90 |  |
| **DSC_0482.jpg** | SSW22-NH5-MOC4-Net3-Pegea socia | *Pegea socia* | FreshSpecimen | SSW22-NH5-MOC4-Net3 | Transversal | DV_Zooid.stolon.angle | 89.22 |  |
| **DSC_0482.jpg** | SSW22-NH5-MOC4-Net3-Pegea socia | *Pegea socia* | FreshSpecimen | SSW22-NH5-MOC4-Net3 | Transversal | DV_Zooid.stolon.angle | 87.5 |  |
