# Supplementary material for: A New Molecular Phylogeny of Salps (Tunicata: Thalicea: Salpida) and the Evolutionary History of Their Colonial Architecture
Source: Integr Org Biol. 2023 Sep 27;5(1):obad037. doi: 10.1093/iob/obad037 (PMC10576244; doi:10.1093/iob/obad037)
Supplement: obad037_Supplemental_Files [file obad037_supplemental_files.zip › SM_Table_1.docx]

SM Table 1. List of the 18S sequence accessions used in the phylogenetic inference analyses.

| Accession number | Species | Sequence type | Collection Date |
| --- | --- | --- | --- |
| OQ863569.1 | *Metcalfina hexagona* | New salp sequence | 14-Sep-2022 |
| OQ863570.1 | *Ihlea punctata* | New salp sequence | 24-Apr-2022 |
| OQ863571.1 | *Ihlea punctata* | New salp sequence | 24-Apr-2022 |
| OQ863572.1 | *Cyclosalpa bakeri* | New salp sequence | 26-Jun-2022 |
| OQ863573.1 | *Iasis cylindrica* | New salp sequence | 22-Apr-2022 |
| OQ863574.1 | *Cyclosalpa polae* | New salp sequence | 14-Sep-2022 |
| OQ863575.1 | *Ritteriella amboinensis* | New salp sequence | 24-Apr-2022 |
| OQ863576.1 | *Cyclosalpa quadriluminis* | New salp sequence | 14-Sep-2022 |
| OQ863577.1 | *Helicosalpa virgula* | New salp sequence | 16-Sep-2022 |
| OQ863578.1 | *Helicosalpa virgula* | New salp sequence | Apr-2021 |
| OQ863579.1 | *Ritteriella amboinensis* | New salp sequence | 27-Jun-2022 |
| OQ863580.1 | *Ihlea punctata* | New salp sequence | 30-Jun-2022 |
| OQ863581.1 | *Helicosalpa younti* | New salp sequence | 26-Jun-2022 |
| OQ863582.1 | *Cyclosalpa pinnata* | New salp sequence | 27-Jun-2022 |
| OQ863583.1 | *Ritteriella retracta* | New salp sequence | 13-Sep-2022 |
| OQ863584.1 | *Ritteriella retracta* | New salp sequence | 13-Sep-2022 |
| FM244864.1 | *Cyclosalpa quadriluminis* | Salp sequence | Retrieved from GenBank |
| FM244865.1 | *Ihlea racovitzai* | Salp sequence | Retrieved from GenBank |
| FM244866.1 | *Iasis cylindrica* | Salp sequence | Retrieved from GenBank |
| FM244867.1 | *Salpa thompsoni* | Salp sequence | Retrieved from GenBank |
| HQ015377.1 | Salpidae gen. nov. sp. nov. A | Salp sequence | Retrieved from GenBank |
| HQ015406.1 | *Salpa thompsoni* | Salp sequence | Retrieved from GenBank |
| HQ015415.1 | *Thalia democratica* | Salp sequence | Retrieved from GenBank |
| HQ015414.1 | *Thalia democratica* | Salp sequence | Retrieved from GenBank |
| HQ015413.1 | *Thalia democratica* | Salp sequence | Retrieved from GenBank |
| HQ015412.1 | *Thalia orientalis* | Salp sequence | Retrieved from GenBank |
| HQ015411.1 | *Ritteriella retracta* | Salp sequence | Retrieved from GenBank |
| HQ015410.1 | *Ritteriella retracta* | Salp sequence | Retrieved from GenBank |
| HQ015409.1 | *Salpa fusiformis* | Salp sequence | Retrieved from GenBank |
| HQ015408.1 | *Salpa maxima* | Salp sequence | Retrieved from GenBank |
| HQ015407.1 | *Salpa maxima* | Salp sequence | Retrieved from GenBank |
| HQ015405.1 | *Salpa aspera* | Salp sequence | Retrieved from GenBank |
| HQ015404.1 | *Brooksia rostrata* | Salp sequence | Retrieved from GenBank |
| HQ015403.1 | *Brooksia rostrata* | Salp sequence | Retrieved from GenBank |
| HQ015402.1 | *Iasis cylindrica* | Salp sequence | Retrieved from GenBank |
| HQ015401.1 | *Iasis cylindrica* | Salp sequence | Retrieved from GenBank |
| HQ015400.1 | *Iasis cylindrica* | Salp sequence | Retrieved from GenBank |
| HQ015399.1 | *Iasis cylindrica* | Salp sequence | Retrieved from GenBank |
| HQ015398.1 | *Cyclosalpa sewelli* | Salp sequence | Retrieved from GenBank |
| HQ015397.1 | *Cyclosalpa quadriluminis* | Salp sequence | Retrieved from GenBank |
| HQ015396.1 | *Cyclosalpa polae* | Salp sequence | Retrieved from GenBank |
| HQ015395.1 | *Cyclosalpa sewelli* | Salp sequence | Retrieved from GenBank |
| HQ015394.1 | *Cyclosalpa polae* | Salp sequence | Retrieved from GenBank |
| HQ015393.1 | *Cyclosalpa floridana* | Salp sequence | Retrieved from GenBank |
| HQ015392.1 | *Cyclosalpa affinis* | Salp sequence | Retrieved from GenBank |
| HQ015391.1 | *Cyclosalpa affinis* | Salp sequence | Retrieved from GenBank |
| HQ015390.1 | *Thetys vagina* | Salp sequence | Retrieved from GenBank |
| HQ015389.1 | *Soestia zonaria* | Salp sequence | Retrieved from GenBank |
| HQ015388.1 | *Pegea bicaudata* | Salp sequence | Retrieved from GenBank |
| HQ015387.1 | *Pegea confoederata* | Salp sequence | Retrieved from GenBank |
| HQ015386.1 | *Pegea confoederata* | Salp sequence | Retrieved from GenBank |
| HQ015378.1 | *Ihlea racovitzai* | Salp sequence | Retrieved from GenBank |
| KR057223.1 | *Brooksia lacromae* | Salp sequence | Retrieved from GenBank |
| KR057222.1 | *Brooksia lacromae* | Salp sequence | Retrieved from GenBank |
| MZ333593.1 | *Salpa younti* | Salp sequence | Retrieved from GenBank |
| AB859889.1 | *Thalia longicauda* | Salp sequence | Retrieved from GenBank |
| AB013011.1 | *Pyrosoma atlanticum* | Outgroup sequence | Retrieved from GenBank |
| AB013012.1 | *Doliolum nationalis* | Outgroup sequence | Retrieved from GenBank |
| AB013017.1 | *Ciona intestinalis* | Outgroup sequence | Retrieved from GenBank |
| D14366.1 | *Thalia democratica* | Outgroup sequence | Retrieved from GenBank |
| FM244861.1 | *Doliolum denticulatum* | Outgroup sequence | Retrieved from GenBank |
| FM244862.1 | *Pyrosoma godeauxi* | Outgroup sequence | Retrieved from GenBank |
| FM244863.1 | *Pyrosomella verticillata* | Outgroup sequence | Retrieved from GenBank |
| L12426.2 | *Molgula manhattensis* | Outgroup sequence | Retrieved from GenBank |
| M91181.1 | *Echinorhinus cookei* | Outgroup sequence | Retrieved from GenBank |
| M97574.1 | *Myxine glutinosa* | Outgroup sequence | Retrieved from GenBank |
| M97571.1 | *Branchiostoma floridae* | Outgroup sequence | Retrieved from GenBank |
| AB013014.1 | *Oikopleura dioica* | Outgroup sequence | Retrieved from GenBank |
| AY903925.1 | *Halocynthia igaboja* | Outgroup sequence | Retrieved from GenBank |
| FM244840.1 | *Clavelina meridionalis* | Outgroup sequence | Retrieved from GenBank |
| FM244841.1 | *Pycnoclavella aff. detorta* | Outgroup sequence | Retrieved from GenBank |
| AB075543.1 | *Megalodicopia hians* | Outgroup sequence | Retrieved from GenBank |
| L12378.2 | *Ascidia ceratodes* | Outgroup sequence | Retrieved from GenBank |
| AJ250778.1 | *Ciona intestinalis* | Outgroup sequence | Retrieved from GenBank |
| AB104873.1 | *Perophora sagamiensis* | Outgroup sequence | Retrieved from GenBank |
| AF165821.2 | *Chelyosoma siboja* | Outgroup sequence | Retrieved from GenBank |
| HQ015385.1 | *Pyrosoma atlanticum* | Outgroup sequence | Retrieved from GenBank |
| HQ015384.1 | *Pyrosoma godeauxi* | Outgroup sequence | Retrieved from GenBank |
| HQ015383.1 | *Pyrosomella verticillata* | Outgroup sequence | Retrieved from GenBank |
| HQ015382.1 | *Pyrosomella verticillata* | Outgroup sequence | Retrieved from GenBank |
| HQ015381.1 | *Pyrosoma atlanticum* | Outgroup sequence | Retrieved from GenBank |
| HQ015380.1 | *Pyrosomella verticillata* | Outgroup sequence | Retrieved from GenBank |
| HQ015379.1 | *Pyrostremma spinosum* | Outgroup sequence | Retrieved from GenBank |
| HQ015376.1 | *Doliolum denticulatum* | Outgroup sequence | Retrieved from GenBank |
