# Supplementary material for: A New Molecular Phylogeny of Salps (Tunicata: Thalicea: Salpida) and the Evolutionary History of Their Colonial Architecture
Source: Integr Org Biol. 2023 Sep 27;5(1):obad037. doi: 10.1093/iob/obad037 (PMC10576244; doi:10.1093/iob/obad037)
Supplement: obad037_Supplemental_Files [file obad037_supplemental_files.zip › SM_Table_2.docx]

SM Table 2. Dorsoventral zooid-stolon angle measurements.

| **Filename** | **Source** | **Species** | **Specimen_type** | **Specimen** | **Architecture** | **Trait** | **State** | **Notes** |
| --- | --- | --- | --- | --- | --- | --- | --- | --- |
| **DSC_0482.jpg** | SSW22-NH5-MOC4-Net3-Pegea socia | *Pegea socia* | FreshSpecimen | 13 | Transversal | DV_Zooid.stolon.angle | 88.60 |  |
| **IMG0007** | Kona April 2022 | *Cyclosalpa affinis* | FreshSpecimen | D15-Caff-B-1 | Whorl | DV_Zooid.stolon.angle | 90.28 |  |
| **IMG0007** | Kona April 2022 | *Cyclosalpa affinis* | FreshSpecimen | D15-Caff-B-1 | Whorl | DV_Zooid.stolon.angle | 86.68 |  |
| **IMG0007** | Kona April 2022 | *Cyclosalpa affinis* | FreshSpecimen | D15-Caff-B-1 | Whorl | DV_Zooid.stolon.angle | 92.28 |  |
| **IMG0016** | Kona April 2022 | *Cyclosalpa polae* | FreshSpecimen | D15-Cpol-B-1 | Cluster | DV_Zooid.stolon.angle | 82.75 |  |
| **IMG0016** | Kona April 2022 | *Cyclosalpa polae* | FreshSpecimen | D15-Cpol-B-1 | Cluster | DV_Zooid.stolon.angle | 85.27 |  |
| **IMG0016** | Kona April 2022 | *Cyclosalpa polae* | FreshSpecimen | D15-Cpol-B-1 | Cluster | DV_Zooid.stolon.angle | 60.00 |  |
| **IMG0062** | Kona April 2022 | *Cyclosalpa sewelli* | FreshSpecimen | D16-Csew-B-1 | Cluster | DV_Zooid.stolon.angle | 78.95 |  |
| **IMG0062** | Kona April 2022 | *Cyclosalpa sewelli* | FreshSpecimen | D16-Csew-B-1 | Cluster | DV_Zooid.stolon.angle | 75.79 |  |
| **IMG0062** | Kona April 2022 | *Cyclosalpa sewelli* | FreshSpecimen | D16-Csew-B-1 | Cluster | DV_Zooid.stolon.angle | 52.58 |  |
| **IMG0089** | Kona April 2022 | *Cyclosalpa affinis* | FreshSpecimen | D17-Caff-B-1 | Whorl | DV_Zooid.stolon.angle | 85.15 |  |
| **IMG0089** | Kona April 2022 | *Cyclosalpa affinis* | FreshSpecimen | D17-Caff-B-1 | Whorl | DV_Zooid.stolon.angle | 86.91 |  |
| **IMG0089** | Kona April 2022 | *Cyclosalpa affinis* | FreshSpecimen | D17-Caff-B-1 | Whorl | DV_Zooid.stolon.angle | 88.00 |  |
| **IMG0097** | Kona April 2022 | *Cyclosalpa affinis* | FreshSpecimen | D17-Caff-B-2 | Whorl | DV_Zooid.stolon.angle | 86.09 |  |
| **IMG0097** | Kona April 2022 | *Cyclosalpa affinis* | FreshSpecimen | D17-Caff-B-2 | Whorl | DV_Zooid.stolon.angle | 89.75 |  |
| **IMG0097** | Kona April 2022 | *Cyclosalpa affinis* | FreshSpecimen | D17-Caff-B-2 | Whorl | DV_Zooid.stolon.angle | 86.62 |  |
| **Img0108** | Kona April 2022 | *Iasis cylindrica* | FreshSpecimen | D18-Icyl-B-1 | Linear | DV_Zooid.stolon.angle | 19.53 |  |
| **Img0108** | Kona April 2022 | *Iasis cylindrica* | FreshSpecimen | D18-Icyl-B-1 | Linear | DV_Zooid.stolon.angle | 16.98 |  |
| **Img0108** | Kona April 2022 | *Iasis cylindrica* | FreshSpecimen | D18-Icyl-B-1 | Linear | DV_Zooid.stolon.angle | 20.13 |  |
| **Img0124** | Kona April 2022 | *Thalia longicauda* | FreshSpecimen | D20-Tlon-B-1 | Oblique | DV_Zooid.stolon.angle | 46.15 |  |
| **Img0124** | Kona April 2022 | *Thalia longicauda* | FreshSpecimen | D20-Tlon-B-1 | Oblique | DV_Zooid.stolon.angle | 50.12 |  |
| **Img0124** | Kona April 2022 | *Thalia longicauda* | FreshSpecimen | D20-Tlon-B-1 | Oblique | DV_Zooid.stolon.angle | 49.30 |  |
| **DSC_0904** | Kona April 2022 | *Cyclosalpa bakeri* | FreshSpecimen | D24-Cbak-B-1 | Whorl | DV_Zooid.stolon.angle | 87.54 |  |
| **DSC_0904** | Kona April 2022 | *Cyclosalpa bakeri* | FreshSpecimen | D24-Cbak-B-1 | Whorl | DV_Zooid.stolon.angle | 89.98 |  |
| **DSC_0904** | Kona April 2022 | *Cyclosalpa bakeri* | FreshSpecimen | D24-Cbak-B-1 | Whorl | DV_Zooid.stolon.angle | 88.47 |  |
| **DSC_0924** | Kona April 2022 | *Soestia zonaria* | FreshSpecimen | D24-Szon-B-1 | Linear | DV_Zooid.stolon.angle | 1.56 |  |
| **DSC_0924** | Kona April 2022 | *Soestia zonaria* | FreshSpecimen | D24-Szon-B-1 | Linear | DV_Zooid.stolon.angle | 1.89 |  |
| **DSC_0924** | Kona April 2022 | *Soestia zonaria* | FreshSpecimen | D24-Szon-B-1 | Linear | DV_Zooid.stolon.angle | 1.84 |  |
| **DSC_0951** | Kona April 2022 | *Salpa fusiformis* | FreshSpecimen | D24-Sfus-B-1 | Linear | DV_Zooid.stolon.angle | 17.80 |  |
| **DSC_0951** | Kona April 2022 | *Salpa fusiformis* | FreshSpecimen | D24-Sfus-B-1 | Linear | DV_Zooid.stolon.angle | 18.77 |  |
| **DSC_0977** | Kona April 2022 | *Cyclosalpa bakeri* | FreshSpecimen | D26-Cbak-B-1 | Whorl | DV_Zooid.stolon.angle | 88.85 |  |
| **DSC_0977** | Kona April 2022 | *Cyclosalpa bakeri* | FreshSpecimen | D26-Cbak-B-1 | Whorl | DV_Zooid.stolon.angle | 85.27 |  |
| **DSC_0977** | Kona April 2022 | *Cyclosalpa bakeri* | FreshSpecimen | D26-Cbak-B-1 | Whorl | DV_Zooid.stolon.angle | 86.44 |  |
| **DSC_0970** | Kona April 2022 | *Pegea sp.* | FreshSpecimen | D25-Psp-B-1 | Transversal | DV_Zooid.stolon.angle | 87.46 |  |
| **DSC_0970** | Kona April 2022 | *Pegea sp.* | FreshSpecimen | D25-Psp-B-1 | Transversal | DV_Zooid.stolon.angle | 88.44 |  |
| **DSC_0970** | Kona April 2022 | *Pegea sp.* | FreshSpecimen | D25-Psp-B-1 | Transversal | DV_Zooid.stolon.angle | 89.02 |  |
| **DSC_0964** | Kona April 2022 | *Cyclosalpa quadriluminis* | FreshSpecimen | D25-Cqua-B-1 | Whorl | DV_Zooid.stolon.angle | 87.38 |  |
| **DSC_0964** | Kona April 2022 | *Cyclosalpa quadriluminis* | FreshSpecimen | D25-Cqua-B-1 | Whorl | DV_Zooid.stolon.angle | 83.80 |  |
| **DSC_0964** | Kona April 2022 | *Cyclosalpa quadriluminis* | FreshSpecimen | D25-Cqua-B-1 | Whorl | DV_Zooid.stolon.angle | 85.64 |  |
| **Img0048** | Kona April 2022 | *Salpa aspera* | FreshSpecimen | D20-Sasp-B-1 | Linear | DV_Zooid.stolon.angle | 19.27 |  |
| **Img0048** | Kona April 2022 | *Salpa aspera* | FreshSpecimen | D20-Sasp-B-1 | Linear | DV_Zooid.stolon.angle | 16.91 |  |
| **Img0048** | Kona April 2022 | *Salpa aspera* | FreshSpecimen | D20-Sasp-B-1 | Linear | DV_Zooid.stolon.angle | 7.50 |  |
| **Img0132** | Kona April 2022 | *Iasis cylindrica* | FreshSpecimen | D22-Icyl-B-2 | Linear | DV_Zooid.stolon.angle | 17.14 |  |
| **Img0132** | Kona April 2022 | *Iasis cylindrica* | FreshSpecimen | D22-Icyl-B-2 | Linear | DV_Zooid.stolon.angle | 20.47 |  |
| **Img0132** | Kona April 2022 | *Iasis cylindrica* | FreshSpecimen | D22-Icyl-B-2 | Linear | DV_Zooid.stolon.angle | 12.76 |  |
| **Img0152** | Kona April 2022 | *Ritteriella amboinensis* | FreshSpecimen | D22-Ramb-B-1 | Bipinnate | DV_Zooid.stolon.angle | 9.84 | lateral view of zooid, used endostyle and tip of viscerae for angle |
| **Img0152** | Kona April 2022 | *Ritteriella amboinensis* | FreshSpecimen | D22-Ramb-B-1 | Bipinnate | DV_Zooid.stolon.angle | 8.56 | lateral view of zooid, used endostyle and tip of viscerae for angle |
| **Img0152** | Kona April 2022 | *Ritteriella amboinensis* | FreshSpecimen | D22-Ramb-B-1 | Bipinnate | DV_Zooid.stolon.angle | 10.97 | lateral view of zooid, used endostyle and tip of viscerae for angle |
| **KonaJJ22-020** | Kona June-July 2022 | *Helicosalpa younti* | FreshSpecimen | D27-Hyou-B-1 | Helical | DV_Zooid.stolon.angle | 82.20 |  |
| **KonaJJ22-020** | Kona June-July 2022 | *Helicosalpa younti* | FreshSpecimen | D27-Hyou-B-1 | Helical | DV_Zooid.stolon.angle | 79.67 |  |
| **KonaJJ22-020** | Kona June-July 2022 | *Helicosalpa younti* | FreshSpecimen | D27-Hyou-B-1 | Helical | DV_Zooid.stolon.angle | 89.92 |  |
| **KonaJJ22-053** | Kona June-July 2022 | *Ritteriella amboinensis* | FreshSpecimen | D27-Ramb-B-1 | Bipinnate | DV_Zooid.stolon.angle | 8.34 | lateral view of zooid, used endostyle and tip of viscerae for angle |
| **KonaJJ22-053** | Kona June-July 2022 | *Ritteriella amboinensis* | FreshSpecimen | D27-Ramb-B-1 | Bipinnate | DV_Zooid.stolon.angle | 9.38 | lateral view of zooid, used endostyle and tip of viscerae for angle |
| **KonaJJ22-053** | Kona June-July 2022 | *Ritteriella amboinensis* | FreshSpecimen | D27-Ramb-B-1 | Bipinnate | DV_Zooid.stolon.angle | 8.30 | lateral view of zooid, used endostyle and tip of viscerae for angle |
| **KonaJJ22-081** | Kona June-July 2022 | *Pegea sp.* | FreshSpecimen | D27-Psp-B-1 | Transversal | DV_Zooid.stolon.angle | 90.06 |  |
| **KonaJJ22-081** | Kona June-July 2022 | *Pegea sp.* | FreshSpecimen | D27-Psp-B-1 | Transversal | DV_Zooid.stolon.angle | 88.86 |  |
| **KonaJJ22-081** | Kona June-July 2022 | *Pegea sp.* | FreshSpecimen | D27-Psp-B-1 | Transversal | DV_Zooid.stolon.angle | 86.56 |  |
| **KonaJJ22-109** | Kona June-July 2022 | *Cyclosalpa sewelli* | FreshSpecimen | D28-Csew-B-1 | Cluster | DV_Zooid.stolon.angle | 85.20 |  |
| **KonaJJ22-109** | Kona June-July 2022 | *Cyclosalpa sewelli* | FreshSpecimen | D28-Csew-B-1 | Cluster | DV_Zooid.stolon.angle | 80.91 | lateral view of zooid, used endostyle and tip of viscerae for angle |
| **KonaJJ22-109** | Kona June-July 2022 | *Cyclosalpa sewelli* | FreshSpecimen | D28-Csew-B-1 | Cluster | DV_Zooid.stolon.angle | 84.90 | lateral view of zooid, used endostyle and tip of viscerae for angle |
| **KonaJJ22-116** | Kona June-July 2022 | *Brooksia rostrata* | FreshSpecimen | D28-Bros-B-1 | Bipinnate | DV_Zooid.stolon.angle | 61.60 | lateral view of zooid, used line between base and tip of the endostyle |
| **KonaJJ22-116** | Kona June-July 2022 | *Brooksia rostrata* | FreshSpecimen | D28-Bros-B-1 | Bipinnate | DV_Zooid.stolon.angle | 59.01 | lateral view of zooid, used line between base and tip of the endostyle |
| **KonaJJ22-116** | Kona June-July 2022 | *Brooksia rostrata* | FreshSpecimen | D28-Bros-B-1 | Bipinnate | DV_Zooid.stolon.angle | 50.16 | lateral view of zooid, used line between base and tip of the endostyle |
| **KonaJJ22-146** | Kona June-July 2022 | *Salpa maxima* | FreshSpecimen | D29-Smax-B-1 | Linear | DV_Zooid.stolon.angle | 15.06 |  |
| **KonaJJ22-146** | Kona June-July 2022 | *Salpa maxima* | FreshSpecimen | D29-Smax-B-1 | Linear | DV_Zooid.stolon.angle | 18.00 |  |
| **KonaJJ22-218** | Kona June-July 2022 | *Cyclosalpa bakeri* | FreshSpecimen | D30-Cbak-B-1 | Whorl | DV_Zooid.stolon.angle | 91.15 |  |
| **KonaJJ22-218** | Kona June-July 2022 | *Cyclosalpa bakeri* | FreshSpecimen | D30-Cbak-B-1 | Whorl | DV_Zooid.stolon.angle | 88.19 |  |
| **KonaJJ22-218** | Kona June-July 2022 | *Cyclosalpa bakeri* | FreshSpecimen | D30-Cbak-B-1 | Whorl | DV_Zooid.stolon.angle | 80.19 | lateral view of zooid, used endostyle and tip of viscerae for angle |
| **KonaJJ22-372** | Kona June-July 2022 | *Ritteriella retracta* | FreshSpecimen | D31-Rsp-B-1 | Bipinnate | DV_Zooid.stolon.angle | 14.43 | lateral view of zooid, used line between base and tip of the endostyle |
| **KonaJJ22-372** | Kona June-July 2022 | *Ritteriella retracta* | FreshSpecimen | D31-Rsp-B-1 | Bipinnate | DV_Zooid.stolon.angle | 10.88 | lateral view of zooid, used line between base and tip of the endostyle |
| **KonaJJ22-372** | Kona June-July 2022 | *Ritteriella retracta* | FreshSpecimen | D31-Rsp-B-1 | Bipinnate | DV_Zooid.stolon.angle | 10.36 | lateral view of zooid, used line between base and tip of the endostyle |
| **KonaJJ22-396** | Kona June-July 2022 | *Cyclosalpa quadriluminis* | FreshSpecimen | D31-Cqua-B-1 | Whorl | DV_Zooid.stolon.angle | 88.36 |  |
| **KonaJJ22-396** | Kona June-July 2022 | *Cyclosalpa quadriluminis* | FreshSpecimen | D31-Cqua-B-1 | Whorl | DV_Zooid.stolon.angle | 89.66 |  |
| **KonaJJ22-396** | Kona June-July 2022 | *Cyclosalpa quadriluminis* | FreshSpecimen | D31-Cqua-B-1 | Whorl | DV_Zooid.stolon.angle | 83.53 |  |
| **KonaJJ22-412** | Kona June-July 2022 | *Salpa maxima* | FreshSpecimen | D31-Smax-B-1 | Linear | DV_Zooid.stolon.angle | 21.60 |  |
| **KonaJJ22-412** | Kona June-July 2022 | *Salpa maxima* | FreshSpecimen | D31-Smax-B-1 | Linear | DV_Zooid.stolon.angle | 19.09 |  |
| **KonaJJ22-408** | Kona June-July 2022 | *Salpa maxima* | FreshSpecimen | D31-Smax-B-1 | Linear | DV_Zooid.stolon.angle | 28.88 |  |
| **KonaJJ22-481** | Kona June-July 2022 | *Pegea sp.* | FreshSpecimen | D31-Psp-B-1 | Transversal | DV_Zooid.stolon.angle | 87.10 |  |
| **KonaJJ22-481** | Kona June-July 2022 | *Pegea sp.* | FreshSpecimen | D31-Psp-B-1 | Transversal | DV_Zooid.stolon.angle | 89.76 |  |
| **KonaJJ22-481** | Kona June-July 2022 | *Pegea sp.* | FreshSpecimen | D31-Psp-B-1 | Transversal | DV_Zooid.stolon.angle | 87.68 |  |
| **KonaJJ22-494** | Kona June-July 2022 | *Ihlea punctata* | FreshSpecimen | D32-Ipun-B-1 | Linear | DV_Zooid.stolon.angle | 23.11 |  |
| **KonaJJ22-494** | Kona June-July 2022 | *Ihlea punctata* | FreshSpecimen | D32-Ipun-B-1 | Linear | DV_Zooid.stolon.angle | 25.13 |  |
| **KonaJJ22-494** | Kona June-July 2022 | *Ihlea punctata* | FreshSpecimen | D32-Ipun-B-1 | Linear | DV_Zooid.stolon.angle | 28.85 |  |
| **KonaJJ22-525** | Kona June-July 2022 | *Iasis cylindrica* | FreshSpecimen | D32-Icyl-B-3 | Linear | DV_Zooid.stolon.angle | 24.01 | young chain |
| **KonaJJ22-525** | Kona June-July 2022 | *Iasis cylindrica* | FreshSpecimen | D32-Icyl-B-3 | Linear | DV_Zooid.stolon.angle | 26.97 | young chain |
| **KonaJJ22-525** | Kona June-July 2022 | *Iasis cylindrica* | FreshSpecimen | D32-Icyl-B-3 | Linear | DV_Zooid.stolon.angle | 29.15 | young chain |
| **KonaJJ22-551** | Kona June-July 2022 | *Ritteriella retracta* | FreshSpecimen | D32-Rsp-B-1 | Bipinnate | DV_Zooid.stolon.angle | 13.22 | lateral view of zooid, used line between base and tip of the endostyle |
| **KonaJJ22-551** | Kona June-July 2022 | *Ritteriella retracta* | FreshSpecimen | D32-Rsp-B-1 | Bipinnate | DV_Zooid.stolon.angle | 11.16 | lateral view of zooid, used line between base and tip of the endostyle |
| **KonaJJ22-551** | Kona June-July 2022 | *Ritteriella retracta* | FreshSpecimen | D32-Rsp-B-1 | Bipinnate | DV_Zooid.stolon.angle | 7.66 | lateral view of zooid, used line between base and tip of the endostyle |
| **KonaJJ22-625** | Kona September 2022 | *Brooksia rostrata* | FreshSpecimen | D33-Bros-B-1 | Bipinnate | DV_Zooid.stolon.angle | 51.78 | lateral view of zooid, used line between base and tip of the endostyle |
| **KonaJJ22-625** | Kona September 2022 | *Brooksia rostrata* | FreshSpecimen | D33-Bros-B-1 | Bipinnate | DV_Zooid.stolon.angle | 55.89 | lateral view of zooid, used line between base and tip of the endostyle |
| **KonaJJ22-625** | Kona September 2022 | *Brooksia rostrata* | FreshSpecimen | D33-Bros-B-1 | Bipinnate | DV_Zooid.stolon.angle | 52.18 | lateral view of zooid, used line between base and tip of the endostyle |
| **Kona09-22-700** | Kona September 2022 | *Cyclosalpa quadriluminis* | FreshSpecimen | D36-Cqua-B-1 | Whorl | DV_Zooid.stolon.angle | 84.70 | used viscera for zooid axis |
| **Kona09-22-700** | Kona September 2022 | *Cyclosalpa quadriluminis* | FreshSpecimen | D36-Cqua-B-1 | Whorl | DV_Zooid.stolon.angle | 89.97 | used viscera for zooid axis |
| **Kona09-22-700** | Kona September 2022 | *Cyclosalpa quadriluminis* | FreshSpecimen | D36-Cqua-B-1 | Whorl | DV_Zooid.stolon.angle | 87.58 | used viscera for zooid axis |
| **Kona09-22-717** | Kona September 2022 | *Soestia zonaria* | FreshSpecimen | D37-Szon-B-1 | Linear | DV_Zooid.stolon.angle | 3.14 |  |
| **Kona09-22-717** | Kona September 2022 | *Soestia zonaria* | FreshSpecimen | D37-Szon-B-1 | Linear | DV_Zooid.stolon.angle | 0.29 |  |
| **Kona09-22-717** | Kona September 2022 | *Soestia zonaria* | FreshSpecimen | D37-Szon-B-1 | Linear | DV_Zooid.stolon.angle | 0.18 |  |
| **Kona09-22-787** | Kona September 2022 | *Metcalfina hexagona* | FreshSpecimen | D39-Mhex-B-1 | Linear | DV_Zooid.stolon.angle | 16.15 |  |
| **Kona09-22-787** | Kona September 2022 | *Metcalfina hexagona* | FreshSpecimen | D39-Mhex-B-1 | Linear | DV_Zooid.stolon.angle | 15.60 |  |
| **Kona09-22-787** | Kona September 2022 | *Metcalfina hexagona* | FreshSpecimen | D39-Mhex-B-1 | Linear | DV_Zooid.stolon.angle | 13.15 |  |
| **Kona09-22-808** | Kona September 2022 | *Cyclosalpa polae* | FreshSpecimen | D40-Cpol-B-1 | Cluster | DV_Zooid.stolon.angle | 52.76 | used viscera for zooid axis |
| **Kona09-22-808** | Kona September 2022 | *Cyclosalpa polae* | FreshSpecimen | D40-Cpol-B-1 | Cluster | DV_Zooid.stolon.angle | 71.27 | used viscera for zooid axis |
| **Kona09-22-808** | Kona September 2022 | *Cyclosalpa polae* | FreshSpecimen | D40-Cpol-B-1 | Cluster | DV_Zooid.stolon.angle | 68.78 | used viscera for zooid axis |
| **Kona09-22-848** | Kona September 2022 | *Brooksia rostrata* | FreshSpecimen | D41-Bros-B-1 | Bipinnate | DV_Zooid.stolon.angle | 19.71 | lateral view of zooid, used line between base and tip of the endostyle |
| **Kona09-22-848** | Kona September 2022 | *Brooksia rostrata* | FreshSpecimen | D41-Bros-B-1 | Bipinnate | DV_Zooid.stolon.angle | 23.48 | lateral view of zooid, used line between base and tip of the endostyle |
| **Kona09-22-848** | Kona September 2022 | *Brooksia rostrata* | FreshSpecimen | D41-Bros-B-1 | Bipinnate | DV_Zooid.stolon.angle | 20.91 | lateral view of zooid, used line between base and tip of the endostyle |
| **Kona09-22-838** | Kona September 2022 | *Iasis cylindrica* | FreshSpecimen | D40-Icyl-B-1 | Linear | DV_Zooid.stolon.angle | 26.09 |  |
| **Kona09-22-838** | Kona September 2022 | *Iasis cylindrica* | FreshSpecimen | D40-Icyl-B-1 | Linear | DV_Zooid.stolon.angle | 29.63 |  |
| **Kona09-22-838** | Kona September 2022 | *Iasis cylindrica* | FreshSpecimen | D40-Icyl-B-1 | Linear | DV_Zooid.stolon.angle | 24.23 |  |
| **Kona09-22-826** | Kona September 2022 | *Salpa maxima* | FreshSpecimen | D40-Smax-B-1 | Linear | DV_Zooid.stolon.angle | 22.50 |  |
| **Kona09-22-826** | Kona September 2022 | *Salpa maxima* | FreshSpecimen | D40-Smax-B-1 | Linear | DV_Zooid.stolon.angle | 33.05 |  |
| **Kona09-22-826** | Kona September 2022 | *Salpa maxima* | FreshSpecimen | D40-Smax-B-1 | Linear | DV_Zooid.stolon.angle | 24.91 |  |
| **Kona09-22-914** | Kona September 2022 | *Helicosalpa virgula* | FreshSpecimen | D43-Hvir-B-1 | Helical | DV_Zooid.stolon.angle | 89.44 |  |
| **Kona09-22-914** | Kona September 2022 | *Helicosalpa virgula* | FreshSpecimen | D43-Hvir-B-1 | Helical | DV_Zooid.stolon.angle | 89.79 |  |
| **Kona09-22-914** | Kona September 2022 | *Helicosalpa virgula* | FreshSpecimen | D43-Hvir-B-1 | Helical | DV_Zooid.stolon.angle | 87.55 |  |
| **Kona09-22-887** | Kona September 2022 | *Thalia longicauda* | FreshSpecimen | D42-Tlon-B-1 | Oblique | DV_Zooid.stolon.angle | 55.87 |  |
| **Kona09-22-887** | Kona September 2022 | *Thalia longicauda* | FreshSpecimen | D42-Tlon-B-1 | Oblique | DV_Zooid.stolon.angle | 56.27 |  |
| **Kona09-22-887** | Kona September 2022 | *Thalia longicauda* | FreshSpecimen | D42-Tlon-B-1 | Oblique | DV_Zooid.stolon.angle | 47.59 |  |
| **KonaMay23_0049** | Kona May 2023 | *Pegea sp.* | FreshSpecimen | D46-Psp-B-1 | Transversal | DV_Zooid.stolon.angle | 88.07 | young zooids |
| **KonaMay23_0049** | Kona May 2023 | *Pegea sp.* | FreshSpecimen | D46-Psp-B-1 | Transversal | DV_Zooid.stolon.angle | 88.29 | young zooids |
| **KonaMay23_0049** | Kona May 2023 | *Pegea sp.* | FreshSpecimen | D46-Psp-B-1 | Transversal | DV_Zooid.stolon.angle | 85.85 | young zooids |
| **KonaMay23_0040** | Kona May 2023 | *Soestia zonaria* | FreshSpecimen | D47-Szon-B-1 | Linear | DV_Zooid.stolon.angle | 3.55 | dead post anesthetic, insides shrivelled |
| **KonaMay23_0040** | Kona May 2023 | *Soestia zonaria* | FreshSpecimen | D47-Szon-B-1 | Linear | DV_Zooid.stolon.angle | 1.06 | dead post anesthetic, insides shrivelled |
| **KonaMay23_0040** | Kona May 2023 | *Soestia zonaria* | FreshSpecimen | D47-Szon-B-1 | Linear | DV_Zooid.stolon.angle | 1.46 | dead post anesthetic, insides shrivelled |
| **KonaMay23_0090** | Kona May 2023 | *Cyclosalpa polae* | FreshSpecimen | D48-Cpol-B-1 | Cluster | DV_Zooid.stolon.angle | 89.30 |  |
| **KonaMay23_0090** | Kona May 2023 | *Cyclosalpa polae* | FreshSpecimen | D48-Cpol-B-1 | Cluster | DV_Zooid.stolon.angle | 89.19 |  |
| **KonaMay23_0090** | Kona May 2023 | *Cyclosalpa polae* | FreshSpecimen | D48-Cpol-B-1 | Cluster | DV_Zooid.stolon.angle | 88.64 | lateral view of zooid, used endostyle and tip of viscerae for angle |
| **KonaMay23_0068** | Kona May 2023 | *Thalia cicar* | FreshSpecimen | D48-Tcic-B-2 | Oblique | DV_Zooid.stolon.angle | 47.66 |  |
| **KonaMay23_0068** | Kona May 2023 | *Thalia cicar* | FreshSpecimen | D48-Tcic-B-2 | Oblique | DV_Zooid.stolon.angle | 48.05 |  |
| **KonaMay23_0068** | Kona May 2023 | *Thalia cicar* | FreshSpecimen | D48-Tcic-B-2 | Oblique | DV_Zooid.stolon.angle | 49.04 |  |
| **KonaMay23_0105** | Kona May 2023 | *Cyclosalpa polae* | FreshSpecimen | D51-Cpol-B-3 | Cluster | DV_Zooid.stolon.angle | 88.27 |  |
| **KonaMay23_0105** | Kona May 2023 | *Cyclosalpa polae* | FreshSpecimen | D51-Cpol-B-3 | Cluster | DV_Zooid.stolon.angle | 87.03 | lateral view of zooid, used endostyle and tip of viscerae for angle |
| **KonaMay23_0105** | Kona May 2023 | *Cyclosalpa polae* | FreshSpecimen | D51-Cpol-B-3 | Cluster | DV_Zooid.stolon.angle | 89.19 | lateral view of zooid, used endostyle and tip of viscerae for angle |
| **D27-Cpin-B-1.mp4 07:32:44:20** | Kona June-July 2022 | *Cyclosalpa pinnata* | FreshSpecimen | D27-Cpin-B-1 (GoPro) | Cluster | DV_Zooid.stolon.angle | 89.59 | Used viscera for zooid axis |
| **D27-Cpin-B-1.mp4 07:32:44:20** | Kona June-July 2022 | *Cyclosalpa pinnata* | FreshSpecimen | D27-Cpin-B-1 (GoPro) | Cluster | DV_Zooid.stolon.angle | 86.13 | Used viscera for zooid axis |
| **D27-Cpin-B-1.mp4 07:32:44:20** | Kona June-July 2022 | *Cyclosalpa pinnata* | FreshSpecimen | D27-Cpin-B-1 (GoPro) | Cluster | DV_Zooid.stolon.angle | 89.25 | Used viscera for zooid axis |
| **tape3_scale41_14 00:02** | LJL Vegas Clips | *Salpa thompsoni* | FreshSpecimen | Vegas tape3_scale41_14 | Linear | DV_Zooid.stolon.angle | 18.44 |  |
| **tape3_scale41_14 00:02** | LJL Vegas Clips | *Salpa thompsoni* | FreshSpecimen | Vegas tape3_scale41_14 | Linear | DV_Zooid.stolon.angle | 17.09 |  |
| **tape3_scale41_14 00:02** | LJL Vegas Clips | *Salpa thompsoni* | FreshSpecimen | Vegas tape3_scale41_14 | Linear | DV_Zooid.stolon.angle | 17.65 |  |
| **Thetys vagina - Salp Chain / Carmel River - 9/14/14 00:24** | <https://vimeo.com/106143426> | *Thetys vagina* | UWvideo | 16 | Oblique | DV_Zooid.stolon.angle | 43.27 |  |
| **Thetys vagina - Salp Chain / Carmel River - 9/14/14 00:24** | <https://vimeo.com/106143426> | *Thetys vagina* | UWvideo | 16 | Oblique | DV_Zooid.stolon.angle | 40.96 |  |
| **Thetys vagina - Salp Chain / Carmel River - 9/14/14 00:24** | <https://vimeo.com/106143426> | *Thetys vagina* | UWvideo | 16 | Oblique | DV_Zooid.stolon.angle | 37.23 |  |
| **Shale_Thalia_democratica_1** | <https://www.naturepl.com/stock-photo--thalia-democratica-salp-chain-deep-sea-atlantic-image01126842.html> | *Thalia democratica* | UWphoto | 3 | Oblique | DV_Zooid.stolon.angle | 64.25 |  |
| **Shale_Thalia_democratica_1** | <https://www.naturepl.com/stock-photo--thalia-democratica-salp-chain-deep-sea-atlantic-image01126842.html> | *Thalia democratica* | UWphoto | 3 | Oblique | DV_Zooid.stolon.angle | 63.93 |  |
| **Shale_Thalia_democratica_1** | <https://www.naturepl.com/stock-photo--thalia-democratica-salp-chain-deep-sea-atlantic-image01126842.html> | *Thalia democratica* | UWphoto | 3 | Oblique | DV_Zooid.stolon.angle | 56.16 |  |
| [**https://scontent.fhio3-1.fna.fbcdn.net/v/t1.6435-9/97992579_2458196494426958_3526566602721984512_n.jpg?_nc_cat=110&ccb=1-7&_nc_sid=730e14&_nc_ohc=axtnPRePZpoAX9flyaX&_nc_ht=scontent.fhio3-1.fna&oh=00_AfDleilkU4rH0gAz1rw9hmNopKKBt-K3ny7iOWimVWtqKw&oe=64987DC8**](https://scontent.fhio3-1.fna.fbcdn.net/v/t1.6435-9/97992579_2458196494426958_3526566602721984512_n.jpg?_nc_cat=110&ccb=1-7&_nc_sid=730e14&_nc_ohc=axtnPRePZpoAX9flyaX&_nc_ht=scontent.fhio3-1.fna&oh=00_AfDleilkU4rH0gAz1rw9hmNopKKBt-K3ny7iOWimVWtqKw&oe=64987DC8) | <https://www.facebook.com/blackwaterdive/posts/seacam1970-aggregate-zooid-of-pegea-bicaudata-its-common-species-off-shore-of-ki/2457547687825172/> | *Pegea bicaudata* | UWphoto | 17 | Transversal | DV_Zooid.stolon.angle | 88.64 |  |
| [**https://scontent.fhio3-1.fna.fbcdn.net/v/t1.6435-9/97992579_2458196494426958_3526566602721984512_n.jpg?_nc_cat=110&ccb=1-7&_nc_sid=730e14&_nc_ohc=axtnPRePZpoAX9flyaX&_nc_ht=scontent.fhio3-1.fna&oh=00_AfDleilkU4rH0gAz1rw9hmNopKKBt-K3ny7iOWimVWtqKw&oe=64987DC8**](https://scontent.fhio3-1.fna.fbcdn.net/v/t1.6435-9/97992579_2458196494426958_3526566602721984512_n.jpg?_nc_cat=110&ccb=1-7&_nc_sid=730e14&_nc_ohc=axtnPRePZpoAX9flyaX&_nc_ht=scontent.fhio3-1.fna&oh=00_AfDleilkU4rH0gAz1rw9hmNopKKBt-K3ny7iOWimVWtqKw&oe=64987DC8) | <https://www.facebook.com/blackwaterdive/posts/seacam1970-aggregate-zooid-of-pegea-bicaudata-its-common-species-off-shore-of-ki/2457547687825172/> | *Pegea bicaudata* | UWphoto | 17 | Transversal | DV_Zooid.stolon.angle | 87.50 |  |
| [**https://scontent.fhio3-1.fna.fbcdn.net/v/t1.6435-9/97992579_2458196494426958_3526566602721984512_n.jpg?_nc_cat=110&ccb=1-7&_nc_sid=730e14&_nc_ohc=axtnPRePZpoAX9flyaX&_nc_ht=scontent.fhio3-1.fna&oh=00_AfDleilkU4rH0gAz1rw9hmNopKKBt-K3ny7iOWimVWtqKw&oe=64987DC8**](https://scontent.fhio3-1.fna.fbcdn.net/v/t1.6435-9/97992579_2458196494426958_3526566602721984512_n.jpg?_nc_cat=110&ccb=1-7&_nc_sid=730e14&_nc_ohc=axtnPRePZpoAX9flyaX&_nc_ht=scontent.fhio3-1.fna&oh=00_AfDleilkU4rH0gAz1rw9hmNopKKBt-K3ny7iOWimVWtqKw&oe=64987DC8) | <https://www.facebook.com/blackwaterdive/posts/seacam1970-aggregate-zooid-of-pegea-bicaudata-its-common-species-off-shore-of-ki/2457547687825172/> | *Pegea bicaudata* | UWphoto | 17 | Transversal | DV_Zooid.stolon.angle | 89.43 |  |
| **DSC_0482.jpg** | SSW22-NH5-MOC4-Net3-Pegea socia | *Pegea socia* | FreshSpecimen | SSW22-NH5-MOC4-Net3 | Transversal | DV_Zooid.stolon.angle | 87.90 |  |
| **DSC_0482.jpg** | SSW22-NH5-MOC4-Net3-Pegea socia | *Pegea socia* | FreshSpecimen | SSW22-NH5-MOC4-Net3 | Transversal | DV_Zooid.stolon.angle | 89.22 |  |
| **DSC_0482.jpg** | SSW22-NH5-MOC4-Net3-Pegea socia | *Pegea socia* | FreshSpecimen | SSW22-NH5-MOC4-Net3 | Transversal | DV_Zooid.stolon.angle | 87.5 |  |
