# Supplementary figures and images for: A New Molecular Phylogeny of Salps (Tunicata: Thalicea: Salpida) and the Evolutionary History of Their Colonial Architecture
Source: Integr Org Biol. 2023 Sep 27;5(1):obad037. doi: 10.1093/iob/obad037 (PMC10576244; doi:10.1093/iob/obad037)

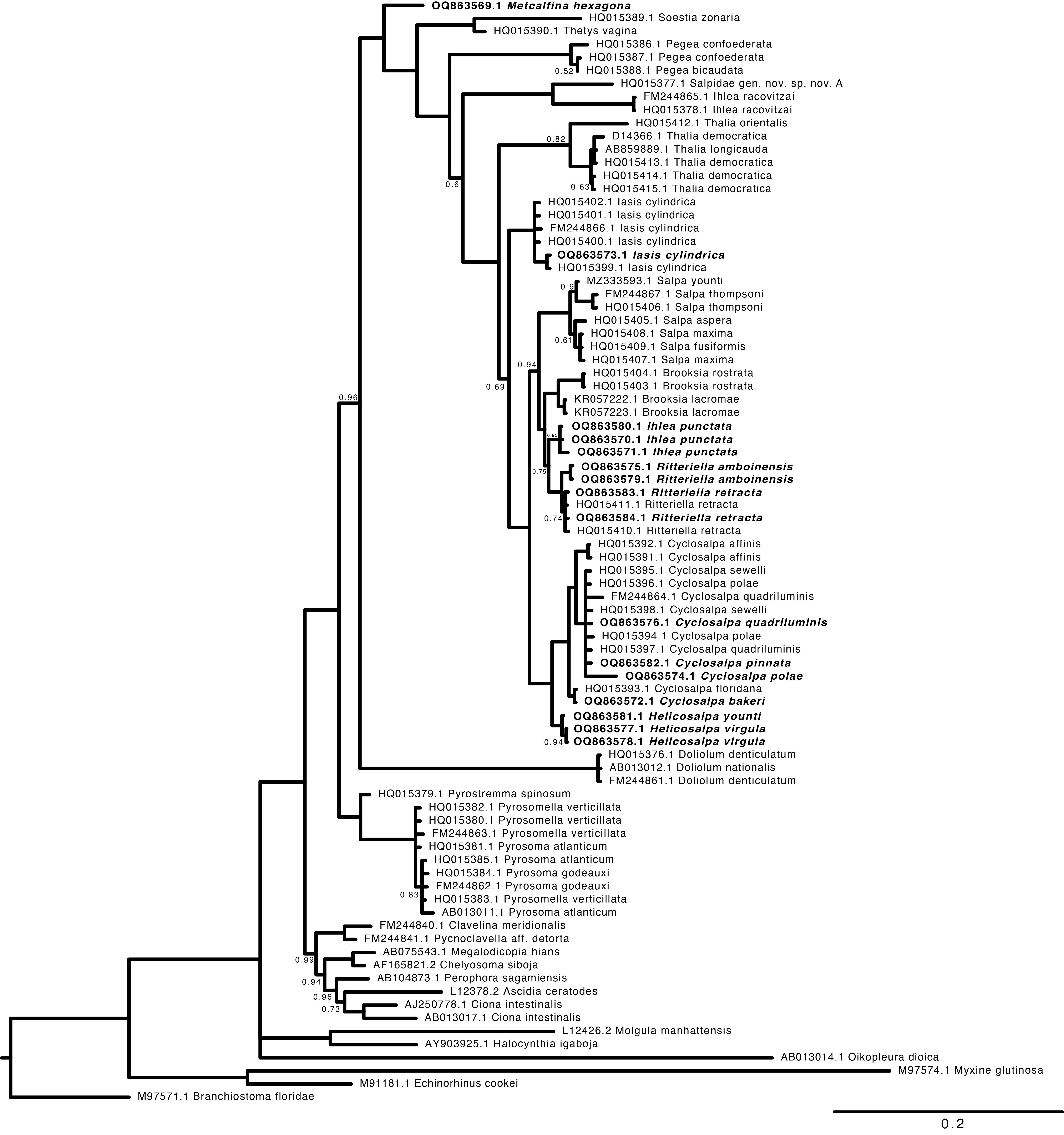

Supplement: obad037_Supplemental_Files [file obad037_supplemental_files.zip › SM_Figure_1.jpg]

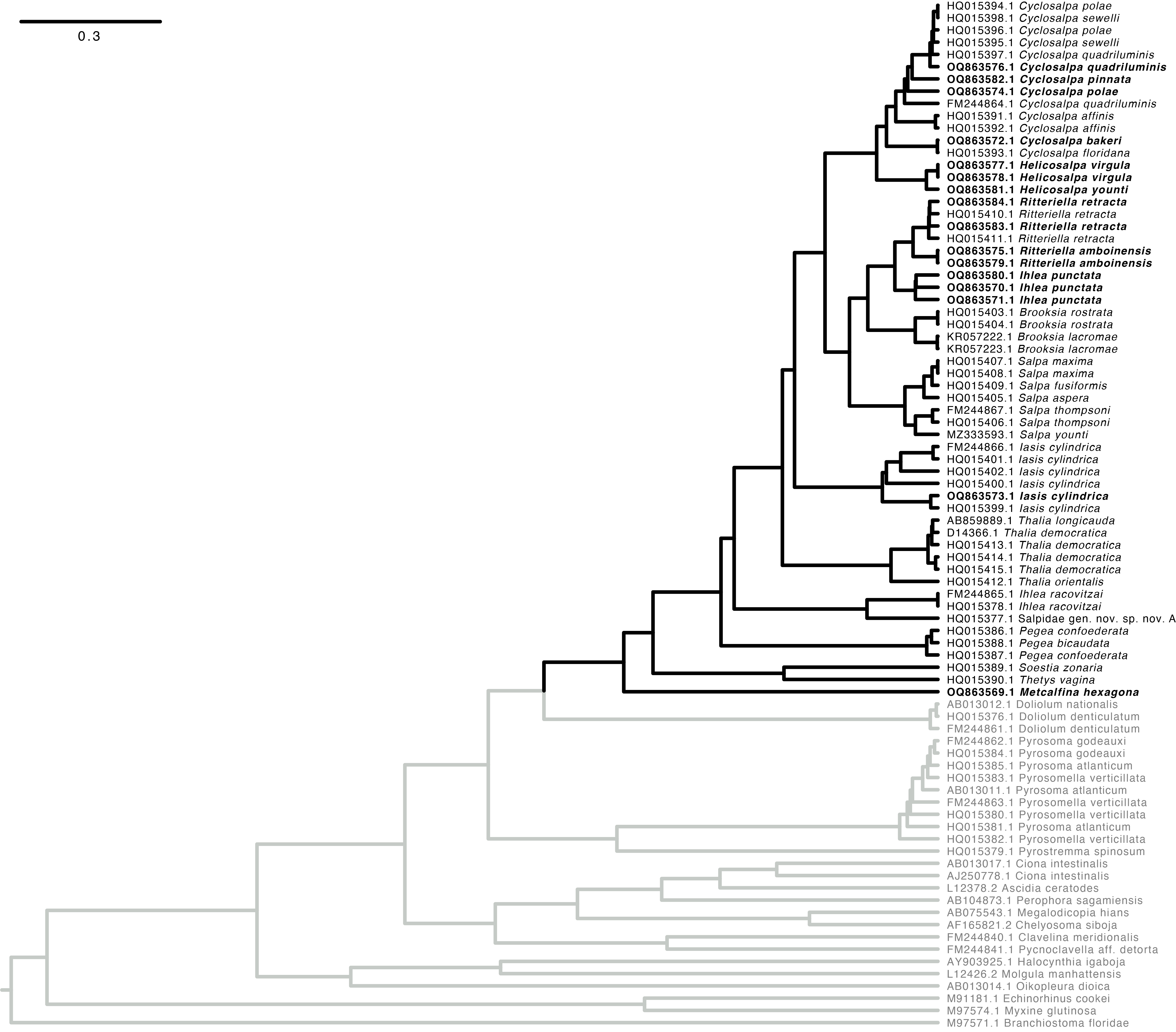

Supplement: obad037_Supplemental_Files [file obad037_supplemental_files.zip › SM_Figure_2.jpg]

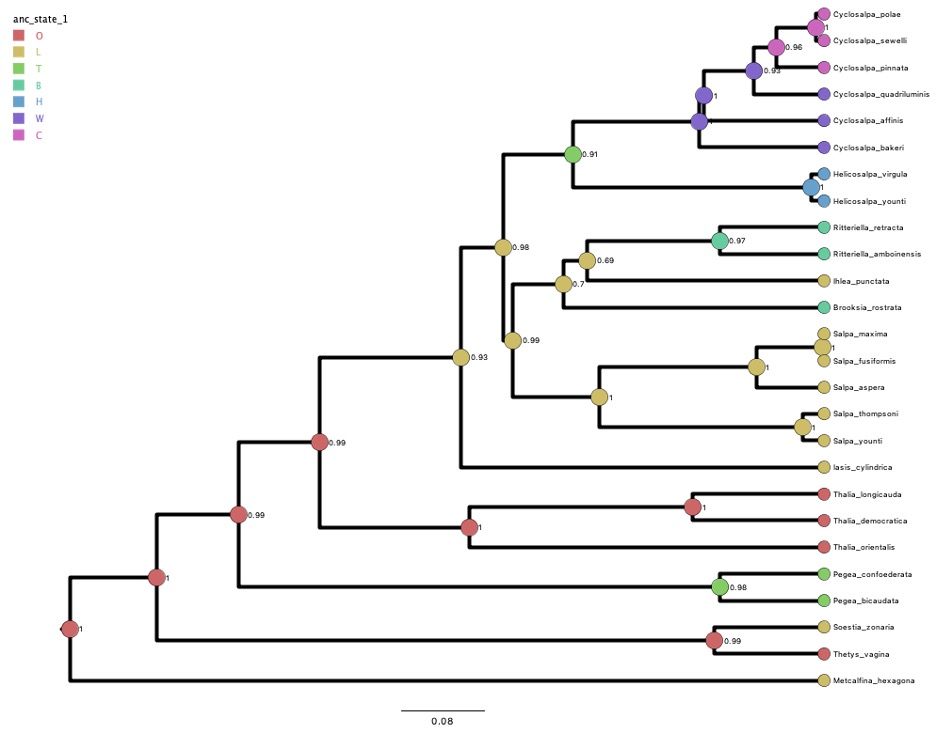

Supplement: obad037_Supplemental_Files [file obad037_supplemental_files.zip › SM_Figure_3.jpg]

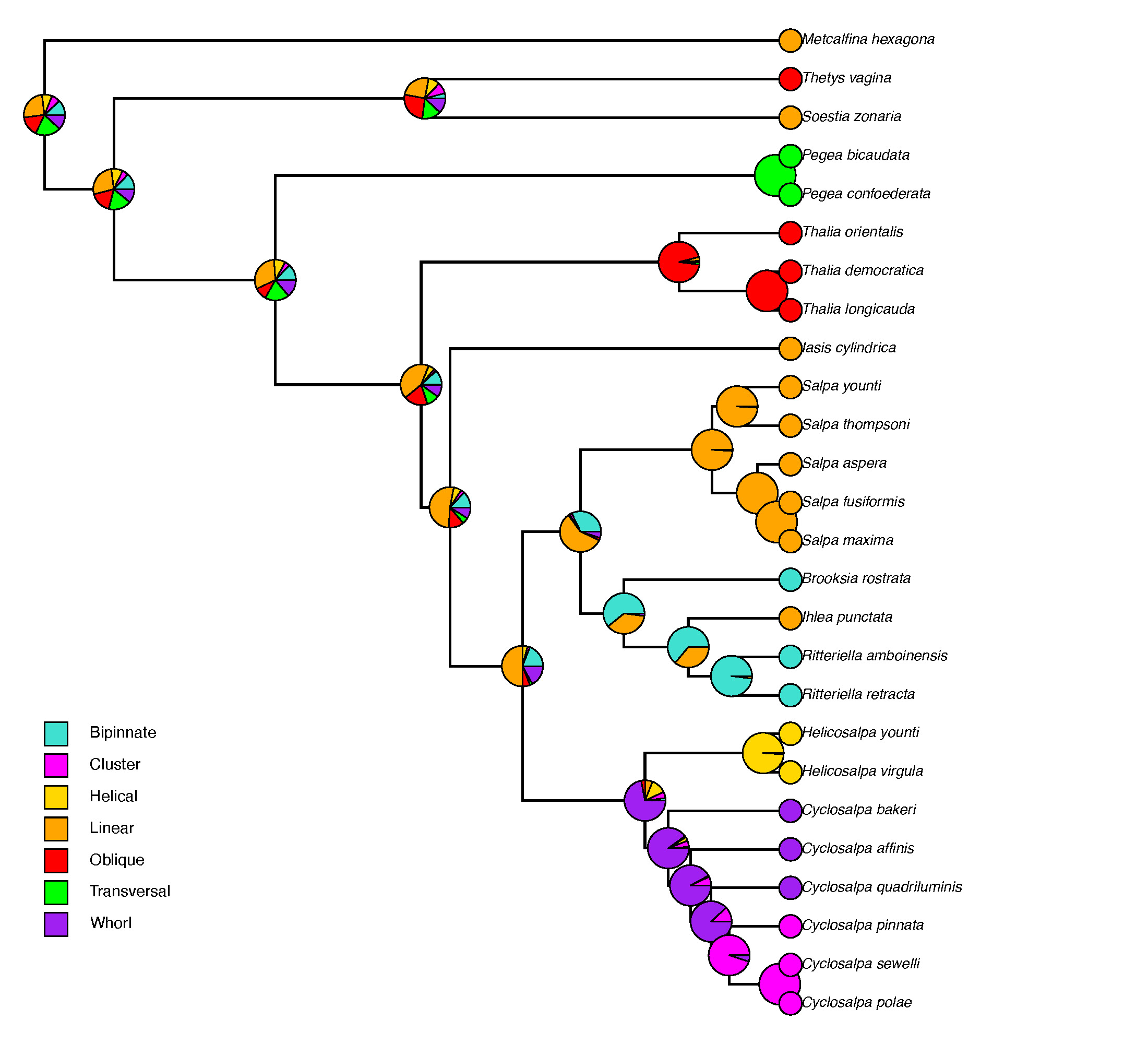

Supplement: obad037_Supplemental_Files [file obad037_supplemental_files.zip › SM_Figure_4.jpg]

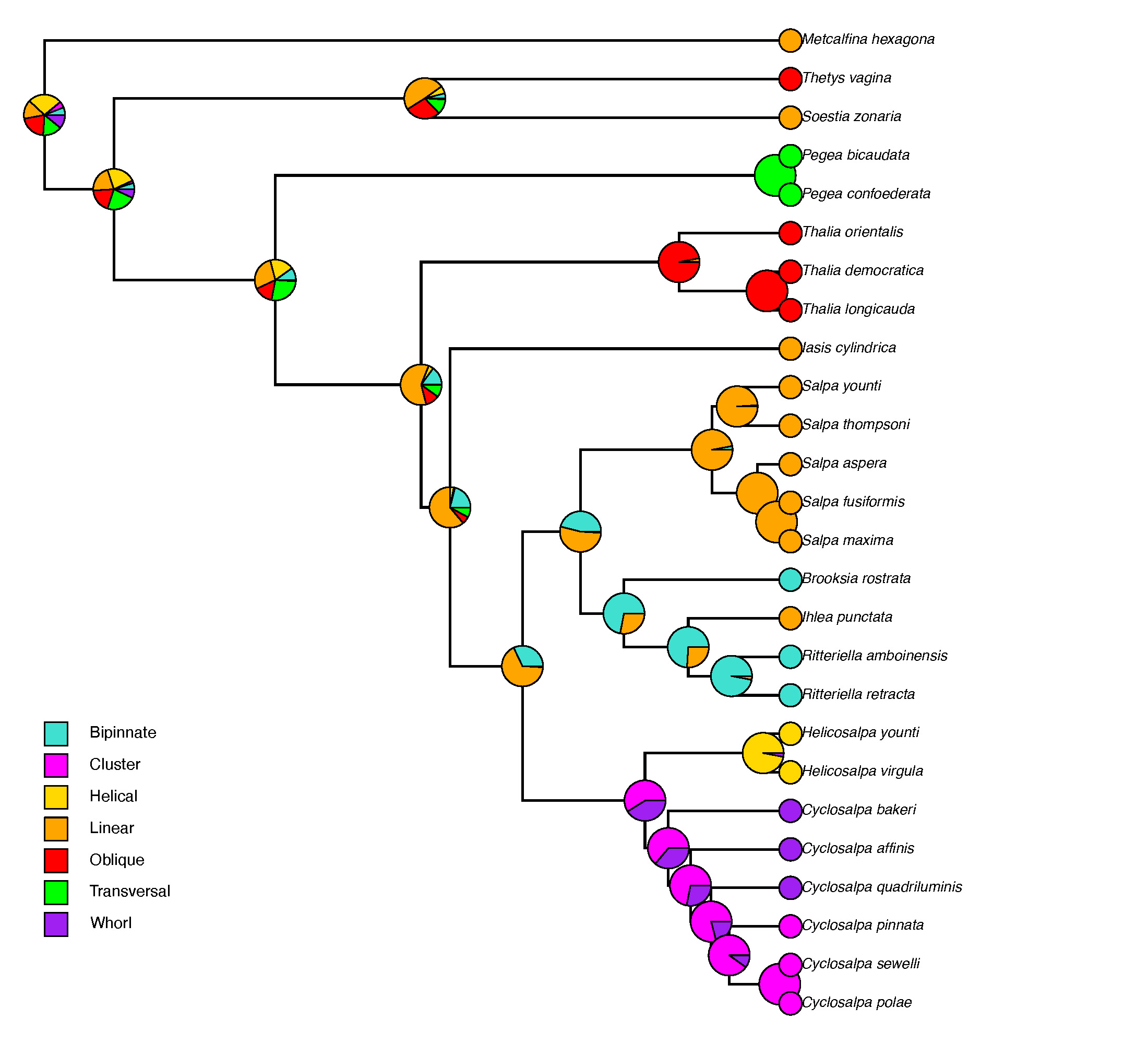

Supplement: obad037_Supplemental_Files [file obad037_supplemental_files.zip › SM_Figure_5.jpg]
